# The syndrome of central hypothyroidism and macroorchidism: IGSF1 controls *TRHR* and *FSHB* expression by differential modulation of pituitary TGFβ and Activin pathways

**DOI:** 10.1038/srep42937

**Published:** 2017-03-06

**Authors:** Marta García, Raquel Barrio, Montserrat García-Lavandeira, Angela R. Garcia-Rendueles, Adela Escudero, Esther Díaz-Rodríguez, Darya Gorbenko Del Blanco, Ana Fernández, Yolanda B. de Rijke, Elena Vallespín, Julián Nevado, Pablo Lapunzina, Vilborg Matre, Patricia M. Hinkle, Anita C. S. Hokken-Koelega, María P. de Miguel, José Manuel Cameselle-Teijeiro, Manuel Nistal, Clara V. Alvarez, José C. Moreno

**Affiliations:** 1 Thyroid Molecular Laboratory, Institute for Medical and Molecular Genetics (INGEMM), La Paz University Hospital, Autonomous University of Madrid, Madrid, Spain; 2 Pediatric Endocrinology and Diabetes, Ramón y Cajal University Hospital, Madrid, Spain; 3 Centro de Investigación en Medicina Molecular y Enfermedades Crónicas (CIMUS), University of Santiago de Compostela, Instituto de Investigación Sanitaria (IDIS), Santiago de Compostela, Spain; 4 Department of Pediatrics, subdivision of Endocrinology, Erasmus Medical Center, Rotterdam, The Netherlands; 5 Department of Clinical Chemistry, Erasmus Medical Center, Rotterdam, The Netherlands; 6 Functional and Structural Genomics, Institute for Medical and Molecular Genetics (INGEMM), La Paz University Hospital, Madrid, Spain; 7 Department of Biosciences, University of Oslo, Oslo, Norway; 8 Department of Pharmacology and Physiology, University of Rochester Medical Center Rochester, NY, USA; 9 Cell Engineering Laboratory, La Paz Hospital Research Institute IdiPAZ, Madrid, Spain; 10 Department of Anatomic Pathology, Medical Faculty, University of Santiago de Compostela, Clinical University Hospital, Santiago de Compostela, Spain; 11 Department of Anatomy, Histology and Neuroscience, Autonomous University of Madrid, Madrid, Spain

## Abstract

*IGSF1 (Immunoglobulin Superfamily* 1) gene defects cause central hypothyroidism and macroorchidism. However, the pathogenic mechanisms of the disease remain unclear. Based on a patient with a full deletion of *IGSF1* clinically followed from neonate to adulthood, we investigated a common pituitary origin for hypothyroidism and macroorchidism, and the role of IGSF1 as regulator of pituitary hormone secretion. The patient showed congenital central hypothyroidism with reduced TSH biopotency, over-secretion of FSH at neonatal minipuberty and macroorchidism from 3 years of age. His markedly elevated inhibin B was unable to inhibit FSH secretion, indicating a status of pituitary inhibin B resistance. We show here that IGSF1 is expressed both in thyrotropes and gonadotropes of the pituitary and in Leydig and germ cells in the testes, but at very low levels in Sertoli cells. Furthermore, IGSF1 stimulates transcription of the thyrotropin-releasing hormone receptor (*TRHR*) by negative modulation of the TGFβ1-Smad signaling pathway, and enhances the synthesis and biopotency of TSH, the hormone secreted by thyrotropes. By contrast, IGSF1 strongly down-regulates the activin-Smad pathway, leading to reduced expression of FSHB, the hormone secreted by gonadotropes. In conclusion, two relevant molecular mechanisms linked to central hypothyroidism and macroorchidism in IGSF1 deficiency are identified, revealing IGSF1 as an important regulator of TGFβ/Activin pathways in the pituitary.

Central Congenital Hypothyroidism (CCH) is a group of hypothalamic-pituitary disorders leading to deficient thyrotropin (TSH) secretion and low thyroid hormone (T4 and T3) synthesis from an otherwise normal thyroid gland^1^,^2^. CCH may coexist with the failure of other pituitary hormones but is rarely associated with non-hormonal clinical features. In 2009, we described the clinical association of familial isolated central hypothyroidism and testicular enlargement, suggesting a genetic nature of the disorder^3^. Recently, such combined (hypophyseal-testicular) phenotype was linked to defects in the *IGSF1* gene in male adolescents and adults^4^. Although, not all IGSF1 deficient patients present macroorchidism^5^.

*IGSF1* gene is located in chromosome Xq26 and encodes a member of the *Immunoglobulin Superfamily* of membrane proteins^6^. IGSF1 contains twelve C2-type immunoglobulin (Ig) loops, a transmembrane domain and a short intracellular C-terminal tail. Despite the presence of Ig loops in its structure, IGSF1 is devoid of independent tyrosine kinase activity^7^. Its function and molecular mechanisms of action are largely unknown. In the past, IGSF1 (also known as InhBP/p120) was proposed as a pituitary receptor for inhibin B, and a regulator of follicle-stimulating hormone (FSH) expression^7^,^8^. However, physical interaction between inhibin B and IGSF1 could not be demonstrated by ligand-receptor binding^9^.

Male and female *Igsf1* knockout mice were reported to have normal phenotype, gonadotropin levels and fertility, leaving a putative role of IGSF1 on the sex hormone axis uncertain^10^. After identification of the human phenotype of IGSF1 deficiency, detailed phenotyping confirmed that *Igsf1*^(−/−)^ mice had reduced TSH pituitary content and serum TSH^4^. However, pituitary Tshb mRNA expression was reported normal. Therefore, the molecular function and implications of IGSF1 on gonadal and thyroid hormone axes remain to be elucidated. Knowing such mechanisms will be valuable to explain the large phenotypic variability of patients with IGSF1 defects and to define the physiological pathways disrupted in the disorder^4^,^11^. Notably, the typical testicular enlargement can be apparently absent in some IGSF1-deficient patients^12^,^13^ while a variable presence of partial deficiency of growth hormone (GH) and prolactin was reported in a few others^4^,^11^. Finally, IGSF1 is present in different tissues, with predominant expression in pituitary and testis^6^,^7^,^14^. However, both tissues are cellularly heterogeneous, and cell type-specific expression of IGSF1 needs to be defined, especially in the pituitary, where contradicting results in rodents (rat and mouse) leave expression of Igsf1 in gonadotropes in the uncertain^4^,^7^.

Here we present the detailed, longitudinal and long-term phenotype (from neonate to adult) of the original patient in whom the disorder was clinically described^3^, harboring a complete deletion in *IGSF1*. We unveiled the cell-type specific expression of the IGSF1 protein in rat pituitary (thyrotropes and gonadotropes) and in human and mice testis (Leydig and Germ cells). We further show that IGSF1 has divergent transcriptional effects on two different pituitary gene promoters. IGSF1 potentiates transcription of the human thyrotropin-releasing hormone receptor (*TRHR*) promoter by repressing the TGFβ1-Smad pathway, a signal which is negatively modulating *TRHR* expression. However, IGSF1 negatively modulates the transcription of the human *FSHB* gene promoter through direct inhibition of the activin-Smad pathway. Clinical, immunohistochemical and molecular correlates of the study suggest that the two main features of the human IGSF1 deficiency may both originate from major pituitary abnormalities. This work unravels a crucial role of IGSF1 as an important regulator of TGFβ superfamily pathways in the pituitary.

## Results

### Clinical case

**Figure 1:**
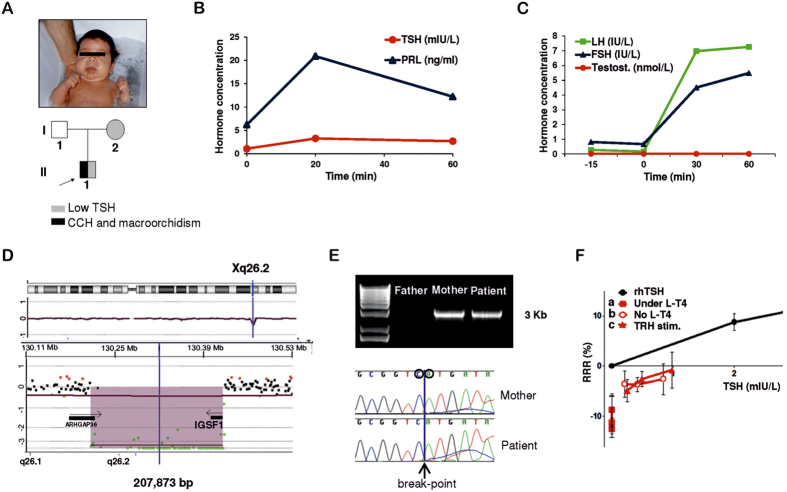
Clinical features and genetic studies of the patient with IGSF1 deletion. (**A**) Picture of the 14 day-old-patient with severe phenotype of hypothyroidism including facial myxedema and protruding tongue. The boy was born to a phenotypically normal father and a euthyroid mother with low TSH. (**B**) TRH stimulation test at 3 years of age. Low TSH response (peak = 3.2 mIU/L) and normal prolactin response consistent with pituitary defect^68^. (**C**) GnRH test at 6 years of age. Abnormal over-stimulation of FSH and LH and unresponsive testosterone without development of clinical signs of precocious puberty, excepting macroorchidism^69^. (**D**) CGH-Array showing the deletion of 207 Kb in Xq26 including the *IGSF1* gene. (**E**) PCR-amplified DNA fragments using primers on the flanking regions of the deletion in non-carrier father, heterozygous mother and hemizygous patient. A band of around 3 Kb is amplifiable when the deletion is present. Precise break-point of deletion as sequenced in PCR fragments from mother and patient. (**F**) TSH bioactivity of patient’s serum at the age of 14 (red) in relation to standard rhTSH (black): (a) patient’s serum under L-T4; (b) patient’s serum after four weeks of levo-thyroxine withdrawal; (c) patient’s serum after TRH stimulation test. Three dilutions for each condition are shown: 1:2, 1:4 and 1:8. CCH: central congenital hypothyroidism, min: minutes, TSH: thyrotropin, PRL: prolactin, FSH: follicle-stimulating hormone, Testost.: testosterone, GnRH: gonadotropin-releasing hormone, rhTSH: recombinant human TSH, L-T4: levothyroxine treatment, TRH stim.: TRH stimulus, RRR: Relative Response Ratio.

The patient is a male of Spanish descent, born to unrelated parents. He was not detected by the TSH-based Neonatal Screening Program, but presented with severe clinical hypothyroidism 14 days after birth, including typical myxedematous face, protruding tongue and lethargy (Fig. 1A). He had low serum free T4 and inappropriately low-normal serum TSH (Table 1). Thyrotropin-releasing hormone (TRH) stimulation test confirmed central hypothyroidism with a poor TSH response and normal prolactin secretion (Fig. 1B). The mother of the patient had abnormally low TSH (0.02 mIU/L) and FT4 in the normal range (19.3 pmol/L). The father showed unremarkable endocrine phenotype (Table 2).

The patient was started on standard levo-thyroxine (L-T4) replacement at 15 days of life. With advancing age, unusually low L-T4 doses were needed to maintain euthyroidism (Table 1), as described in Central Hypothyroidism as compared to Primary Hypothyroidism^15^.

The other pituitary axes were intact in the patient, including normal basal GH levels and adrenocorticotropic hormone (ACTH) dynamic test (Synacthen^®^) (Supplemental Fig. 1A and B). Brain MRI showed normal size and shape of the pituitary. Thyroid ultrasounds and scintigraphy at 6 years of age showed eutopic, mildly hypoplastic thyroid of reduced echogenicity and isotopic uptake (data not shown).

At 14 days of life, patient’s sex hormone profile revealed an abnormal elevation of FSH compared to normal male newborns. FSH continued elevated during “mini-puberty”, a physiological period when the gonadal axis is transiently active^16^,^17^ (Table 1). At the end of mini-puberty (5 months of age) when the axis becomes inactive, patient’s FSH returned to normal levels. Luteinizing hormone (LH) was not elevated and testosterone was normal (Table 1). From 3 years onwards, the patient developed a clearly progressive bilateral and symmetric macroorchidism under normal prepubertal values of FSH, LH, and testosterone (Table 1 and Supplemental Fig. 2). A gonadotropin-releasing hormone (GnRH) test was performed at the age of 6 years showing abnormally increased FSH and LH levels in ratios suggesting initiation of puberty. However, the expected increased in testosterone was not present, ruling out the start of a precocious puberty (Fig. 1C). Testosterone levels remained low until his puberty spontaneously started at a normal age of 12 years, but yet with enlarged testicular volume (12 ml), as measured by orchidometer (Table 1 and Supplemental Fig. 2).

At 14 years, the patient was extensively characterized by an additional TRH test and complete sex and thyroid hormone profiles. L-T4 withdrawal confirmed permanent central hypothyroidism with TSH in the normal ranges and low total and free T4 and T3 (Table 2). A mild improvement in the TSH peak was detectable in response to TRH through age (Supplemental Fig. 1C). Testosterone, estradiol, FSH and the common alpha-subunit (CGA) were normal and LH levels slightly decreased (Table 2). Remarkably, inhibin B and anti-müllerian hormone (AMH) were strongly elevated, indicating an increased number of Sertoli cells in the testis. At this stage, testicular volume was yet 40 ml (normal: 15–25 ml, Supplemental Fig. 2), and final adult volume reached 50 ml (60 × 40 mm, Supplemental Fig. 2), as calculated using a caliper and the Lamber’s formula^18^; testicular ultrasounds showed normal echogenicity, ruling out tumor-related growth. Sperm production was preserved with normal amount (100 million/ml) and mobility of spermatozoids at 15 years of age (data not shown).

### Complete deletion of IGSF1 gene

Direct sequencing of the coding exons and exon/intron boundaries of three candidate genes for central hypothyroidism (*TSHB, CGA* and *TRHR*) revealed no genetic abnormalities.

Comparative Genomic Hybridization (CGH)-Array showed a hemizygous deletion on chromosome X in the patient. The deleted region included the entire *IGSF1* gene sequence (Fig. 1D) and the break-point was studied in the patient and parents using long PCR and Sanger sequencing, revealing a 207.873 Kb deletion in Chr. Xq26.2 (chrX: 130,217,339–130,425,212 GRCh37/hg19), present also in the mother in heterozygote state (Fig. 1E). The whole IGSF1 gene is deleted, including all putative 3′ regulatory regions and the 5′ promoter.

The ARHGAP36 gene is deleted in its 3′-coding and putative 3′regulatory regions. The promoter is conserved. Very little is known about the function and expression pattern of this gene^19^. Its lack of expression has not been linked to any specific animal or human phenotype. Based on the information available, this gene does not seem to have an effect on the phenotype we described, which exactly coincides with that of the typical IGSF1 defect in humans, without the presence of any other additional sub-phenotypes.

### TSH bioactivity is low in IGSF1 deficiency

TSH is one of the pituitary hormones that are glycosylated. This glycosylation is essential for TSH activity. The hypothalamic hormone TRH acts in the pituitary to regulate not only TSH expression but also its correct maturation and bioactivity (glycosylation)^20^,^21^.

Patient’s TSH bioactivity was studied at the age of 14 years from three different serum samples taken: (a) under L-T4 treatment, (b) after four weeks of levo-thyroxine withdrawal prior to a TRH test and (c) 3 hours after TRH stimulation (Fig. 1F). TSH bioactivity was undetectable under L-T4 treatment (a), corresponding to very low immunoreactive TSH levels (Table 2). Four weeks after L-T4 withdrawal (b), serum TSH bioactivity was shown to be significantly decreased despite normal serum TSH quantitative values in the immunoassay (Table 2). TSH bioactivity and quantitative values did not improve after stimulation with TRH (c) (Fig. 1F). This shows that patient’s TSH is not only inappropriately normal, but also that its bioactivity is markedly decreased, suggesting abnormal glycosylation pattern of the TSH^20^. The serum TSH of the patient’s mother (carrier of the IGSF1 deletion) also had decreased bioactivity, whereas the father’s TSH bioactivity was normal (Supplemental Fig. 3A).

### IGSF1 is located in rat pituitary thyrotropes and gonadotropes

**Figure 2:**
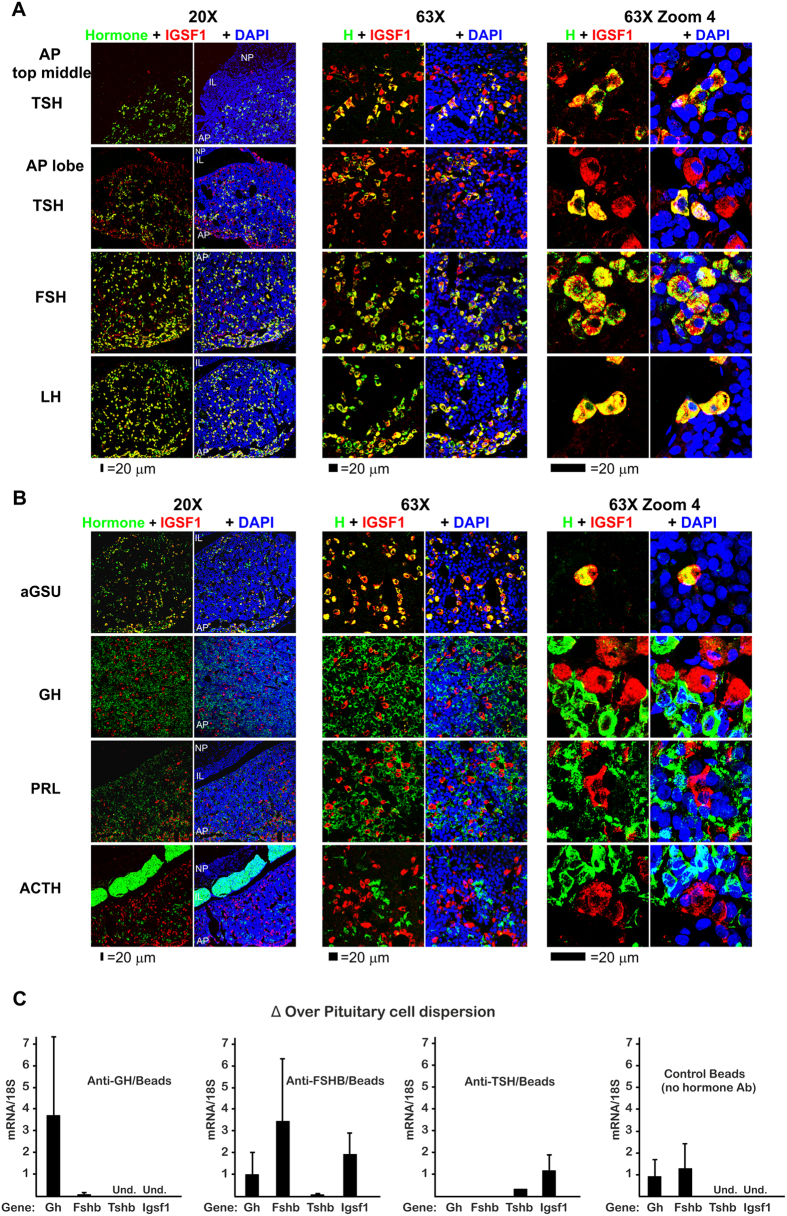
IGSF1 cellular expression in young adult male rat pituitary gland is expressed in thyrotrope and gonadotrope endocrine cells. Coronal pituitary sections were stained and topographical serially studied using confocal microscopy from the middle towards the lobes at different magnifications (20X, 63X and 63XZoom4). A white laser was used to prevent differences in intensity by use of different wavelength lasers. IGSF1 is shown in red pseudocolor while every hormone is shown in green. Nuclei were stained with DAPI and shown in blue. (**A**) IGSF1 is located exclusively in the AP but not in the IL or the NP and co-localizes with the three hormones: TSH beta, FSH beta and LH beta. The number of double positive IGSF1/TSH was higher in the middle of the section (thyrotrope region) than toward the lobes (gonadotrope region). (**B**) All IGSF1 cells co-localize with aGSU, the common alpha subunit for TSH, FSH and LH. No somatotrope (GH), lactotrope (PRL) or corticotrope (ACTH) was found to be expressing IGSF1. Quantifications are shown in Supplemental Fig. 2B. (**C**) Immune-magnetic purification of somatotropes (anti-GH/Beads), gonadotropes (anti-FSHB/Beads) and thyrotropes (anti-TSHB/Beads) from a single cell dispersion of rat pituitary followed by qRT-PCR. Results are expressed as enrichment (gene mRNA/18 S) over the initial cell dispersion. As a control, magnetic beads in the absence of hormone antibody were used. As expected, Gh mRNA was enriched in anti-GH, Fshb mRNA in anti-FSHB and Tshb mRNA in anti-TSHB purified cells. Igsf1 mRNA was detected exclusively in the anti-FSHB and anti-TSHB purified cells. Results are the mean of four independent experiments. AP = adenopituitary. IL = intermediate lobe. NP = neuropituitary.

To evaluate the presence of IGSF1 in the anterior pituitary, western blot in human and rat pituitary extracts and double immunofluorescence with confocal co-localization of rat Igsf1 and each of the pituitary hormones were performed. IGSF1 is abundantly expressed in human and rat adenopituitary by western blot using a commercial antibody, and the major band was of the expected size (around 148 kDa) (Supplemental Fig. 4A). In double immunofluorescence of rat pituitary sections, Igsf1 was co-expressed in all TSH beta positive (+ve) thyrotropes, both in their top-middle and lobe locations of the adenopituitary (Fig. 2A and Supplemental Fig. 4B). Quantitative analysis showed that 90.3% of Igsf1 +ve cells in the pituitary top-middle portion co-expressed TSH in comparison with only 63.9% of the of Igsf1 +ve cells in the lobes. This is consistent with the top-middle pituitary being an area enriched in thyrotropes. Similarly, all gonadotropes (FSH beta +ve or LH beta +ve) expressed Igsf1 in their pituitary lobe location (Fig. 2A and Supplemental Fig. 4B).

Consistent with these results, Igsf1 also co-localized with all cells expressing alpha Glycoprotein Subunit (aGSU), the common subunit for thyrotropic and gonadotropic hormones (Fig. 2B and Supplemental Fig. 4B–D). In contrast, Igsf1 was not present in somatotropes (GH +ve), lactotropes (PRL +ve) or corticotropes (ACTH +ve) (Fig. 2B and Supplemental Fig. 4B). Structurally, although Igsf1 was not an exclusive plasma membrane protein, the Igsf1 staining showed membrane reinforcement and colocalized with E-cadherin indicating a function related to the plasma membrane (Supplemental Fig. 4E). These results are in contrast with previous results by Joustra *et al*. using a different antibody that localized Igsf1 in Pit-1 expressing cells within the rat pituitary, while SF-1/Lh expressing cells did not co-localize with Igsf1^22^.

To confirm the cell-specific pattern of expression of Igsf1 in the pituitary we measured Igsf1 mRNA expression in each type of endocrine cell. We purified enriched populations of somatotropes, gonadotropes and thyrotropes from cell dispersions of young adult male rat pituitary, using antibodies anti-Gh, -Fshb, and -Tshb respectively and immune-magnetic purification (Fig. 2C). qRT-PCR was performed in those purified populations to quantify mRNA expression for *Gh, Fshb, Tshb and Igsf1* together with the control gene 18 s. As expected, in each of the purified populations there was an enrichment of the mRNA for the corresponding hormone: Gh in somatotropes, Fshb in gonadotropes and Tshb in thyrotropes, although this one was less enriched since the number of thyrotrophs in a normal pituitary is less than 10% of the endocrine population^23^,^24^. Igsf1 mRNA was enriched in the gonadotrope and thyrotrope population (Fig. 2C). However, we were unable to detect Igsf1 mRNA in the somatotroph population. This result agrees with the above confocal studies and, together with the western blot, validates our immune co-localization pattern of Igsf1.

### *IGSF1* is mainly present in germ cells and Leydig cells of human and mice adult testis

**Figure 3:**
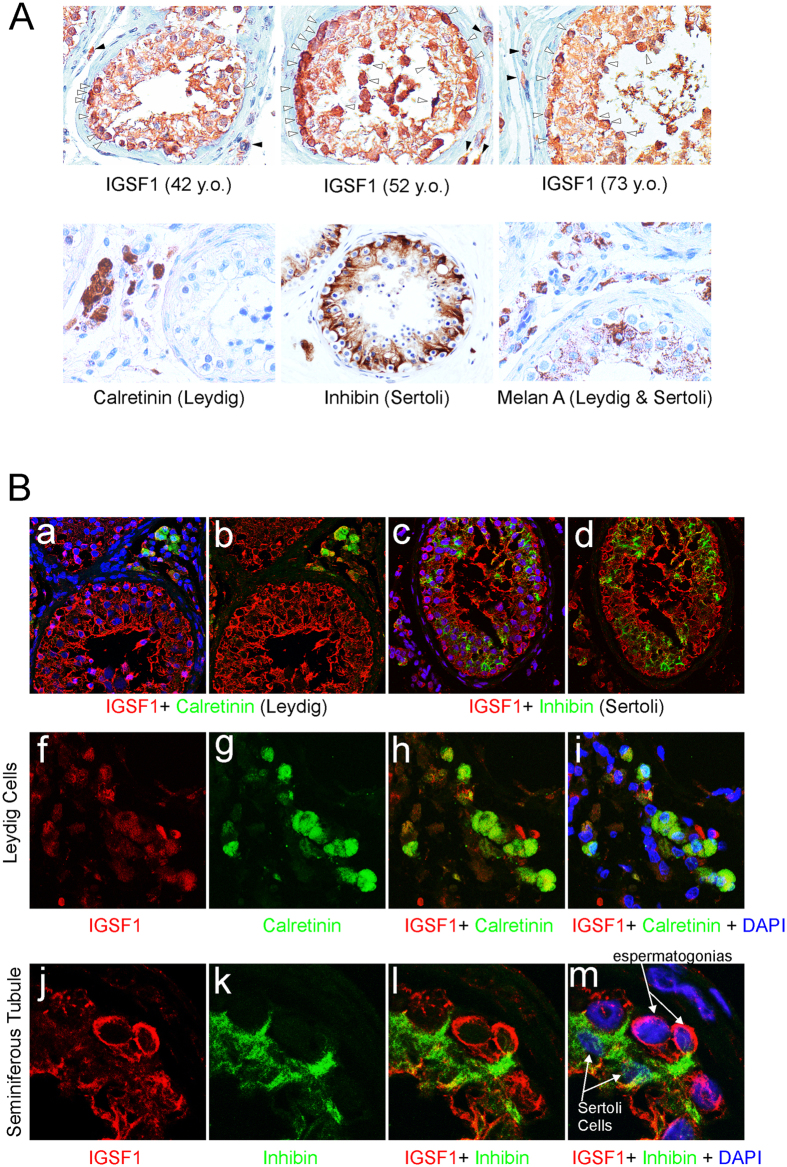
IGSF1 stain germ cells and Leydig cells in human adult testes. (**A**) Immunohistochemistry of IGSF1 in a tissue array of three independent samples of human testes (***a***–***c***, 42, 52, and 73 years old). Staining is seen inside the whole wall of tubules with stronger staining at the basal layer and also at the lumen (white arrowheads). In the interstitium, some peripheral cells are also stained (black arrowheads). The array was also stained for markers of Leydig cells (Calretinin), Sertoli cells (Inhibin) or both (Melan A) (***d***,***e***,***f***, respectively). IGSF1 positive peripheral cells could correspond with Calretinin or Melan A staining outside the tubules. However, inside the tubule, Inhibin or Melan A (both present in Sertoli cells) and IGSF1 do not seem to stain the same populations. (**B**) To further study IGSF1 cell populations double immunofluorescence for IGSF1 + Calretinin (***a***,***b***) and IGSF1 + Inhibin (***c***,***d***) was studied using confocal microscopy. Calretinin (Leydig cells, green) co-localizes with IGSF1 (red) at the periphery of the tubules, although they stain different cell areas. This is further seen when zooming in (***f***–***i***). Opposite, inside the tubule Inhibin does not seem to co-localize with IGSF1 (***c***,***d***). IGSF1 (red) is strong at the periphery and lumen while Inhibin appears in specific areas of the tubule wall as corresponding to its tree-like form. In ***j***–***m*** a zoomed area is observed with two marked spermatogonia stained for IGSF1 (red) and two Sertoli cells stained for Inhibin (green). Sertoli arms surround the IGSF1 positive spermatogonia and are negative for IGSF1. The few yellow spots that could be observed in ***c***,***d*** and ***l***,***m*** may correspond either to crossing points between the two layer of cells (germ cells and Sertoli) or to Sertoli cells expressing IGSF1 only in few and restricted contact areas.

To investigate the presence of IGSF1 in testis, we used immunohistochemistry and double immunofluorescence with confocal co-localization of IGSF1 and three cell-type specific testicular markers (Calretinin, Inhibin and Melan A). Three different samples of human testicular tissue (from donor men of 42, 52 and 73 years of age) were stained for IGSF1 and compared with the typical staining pattern of Leydig cells (Calretinin), Sertoli cells (Inhibin) or both (Melan A) (Fig. 3A). IGSF1 signal was present within and outside the seminiferous tubules. In the interstitium, IGSF1 staining was similar to those typical of Calretinin and Melan A (Fig. 3A*d*,*f*), indicating the presence of IGSF1 in Leydig cells. Within the tubules, IGSF1 stained cells from the basement to the lumen, with reinforced staining of basal and peri-luminal layers (Fig. 3A*a*–*c*). This pattern differs from that typical of Inhibin - strong staining in basal “spikes”, inter-spermatogonia, and no peri-luminal staining - and that of Melan A in the tubules (Fig. 3A*e*,*f*), suggesting that IGSF1 could be absent or have very low expression in human adult Sertoli cells while being present in the germ epithelium.

To further investigate IGSF1 in testicular cell populations, double immunofluorescence for IGSF1 and Calretinin or Inhibin, respectively, was performed. IGSF1 was again detected from basement to lumen within the seminiferous tubules, more intensely at basal and peri-luminal areas (Fig. 3B*a*–*d*). IGSF1 co-localized with Calretinin in Leydig cells, although the staining was less intense than in the tubules (Fig. 3B*a*,*b*,*f*–*i*). Within the seminiferous tubule, double-fluorescence of Inhibin and IGSF1 showed distinct locations and no co-localization, indicating that Sertoli cells are clearly different from the cell population expressing IGSF1 (Fig. 3B*c*,*d*,*j*–*m*) and may not express IGSF1. On the other hand, faint and scarce yellow (co-localization) spot signals could be rarely detected (Fig. 3B*c*,*d*,*l*,*m*). These few yellow spots may correspond either to crossing points between the two layers of cells (germ cells and Sertoli cells) or to Sertoli cells expressing some IGSF1 only in few restricted contact areas with the germ cells.

Therefore, expression of IGSF1 in human adult testis is concentrated mainly the germ cell epithelium, being especially intense in the cytoplasm of spermatogonia. It is also present in Leydig cells and has very low or no expression in adult human Sertoli cells.

Parallel experiments in adult mice testis revealed similar patterns of Igsf1 staining and cell-type specific expression compared to those in human testis (Supplemental Fig. 5), being mainly present in germ cells and Leydig cells. Remarkably, mouse germ epithelium presents a distinct staining of germ granules not found in humans.

### IGSF1 activates the TRHR promoter and represses the FSHB promoter

Our patient presented with reduced TSH secretion combined with reduced serum TSH biopotency. Both processes involve TSH beta (*TSHB*) gene expression and TSH glycosylation respectively, and both are known to be dependent on TRHR activation^20^,^21^. On the other hand, the patient presented with excessive FSH secretion as a neonate which, interestingly, resulted in macroorchidism, as happens in FSH-secreting pituitary adenomas^25^,^26^,^27^. FSH secretion is critically regulated by testicular inhibin B through inhibition of the stimulatory activin-Smad signaling pathway, and IGSF1 was proposed as a putative inhibin B receptor^7^. Based on the evidence that *FSHB* is activated by the activin-Smad pathway^28^,^29^,^30^, the presence of putative Smad-responsive elements in the *FSHB* and *TRHR* promoters was investigated *in silico*. In the human *FSHB* promoter a putative Smad-responsive element (CTGTCTATCTA) at −133 to −123 bp and a putative Smad3-binding element (AGGCAGCCG) at +17 to +25 from transcription start site were identified. In the human *TRHR* promoter a putative Smad3-binding element (AGACAGATA) was identified between positions −1173 and −1165 bp from transcription start site. Although an alteration of the GnRH Receptor could also be involved, it has been demonstrated in rat and mouse models that *Gnrhr* expression in gonadotropes is fully independent of Activin signaling^31^.

**Figure 4:**
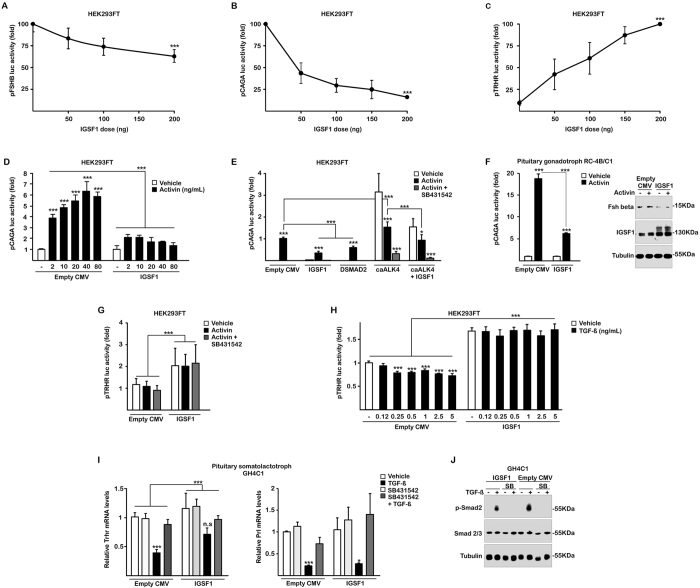
Molecular mechanisms and pathways involved in IGSF1 deficiency. In HEK293FT, IGSF1 represses the activity of both a human *FSHB* −875/+120 minimal promoter (*pFSHB*) (**A**) and its potent surrogate *CAGA-luc* promoter (*pCAGA*)(**B**), in a dose-response manner. (**C**) IGSF1 stimulates basal human *TRHR* promoter (*pTRHR*) activity in a dose-dependent manner. (**D**) A dose-response curve of Activin (2–80 ng/ml) demonstrates the potency of IGSF1 blunting the response to Activin. (**E**) IGSF1 is more potent than a dominant negative SMAD2 mutant (DSMAD2) and is able to repress the constitutive activity of an activating mutant of the Activin receptor (caALK4) in the absence of Activin. SB431542, antagonist of the receptor, was added in all conditions to show the functionality of the pCAGA response, blunted when this inhibitor was present. (**F**) In pituitary gonadotropes, RC-4B/C1, pCAGA response to activin was huge -circa 18 times over control- and reduced to 5 times in the presence of IGSF1 (left). A western blot shows induction of Fshb expression with Activin that was blunted in the presence of IGSF1 (right). (**G**) Activin does not induce the human *TRHR* promoter nor alters the basal stimulation in the presence of IGSF1. (**H**) TGFβ represses the luciferase activity of the *TRHR* promoter in a dose-response manner. Repression is reversed by IGSF1. (**I**) Endogenous expression of Trhr in pituitary GH4C1 cells by qRT-PCR (left). Trhr mRNA is repressed by TGFβ (black bars) and this is reversed by the TGFβ-inhibitor SB431542 (dark grey bars). IGSF1 blocks such repression as efficiently as the inhibitor. Prolactin (Prl) mRNA, another gene repressed by TGFβ, was not affected by the presence of IGSF1 and was equally repressed by TGFβ in its presence. SB431542 blocked TGFβ action in both genes. (**J**) C-terminal phosphorylation of SMAD2 (p-Smad2), mediated by TGFβRII/RI complex upon TGFβ addition, was markedly reduced by IGSF1 in pituitary GH4C1. Extracts were taken after one hour with TGFβ or Vehicle. SB431542, the TGFβRI inhibitor, blocks p-SMAD2 independently of the absence/presence of IGSF1. n.s.: not significant, *p < 0.05, **p < 0.01, ***p < 0.005.

We tested the hypothesis that IGSF1 acts on pituitary regulation of *FSHB* and *TRHR* gene expression. Transfection of increasing concentrations of IGSF1 in the presence of a fixed dose of activin A significantly decreased in a dose-dependent manner the transcriptional activity of a −875 to +120 bp human *FSHB* minimal promoter (minimal *FSHB* promoter) cloned in house (Fig. 4A). We also tested an artificial activin-sensitive promoter (CAGA) containing twelve Smad3-binding motives^32^, previously used in Lbt2 gonadotropic cells yielding a potent transcriptional activity in response to activin, 10-fold higher than that of the *FSHB* promoter^33^. IGSF1 strongly and dose-dependently reduced the activity of the CAGA promoter in presence of a fixed dose of activin A (Fig. 4B). For further experiments CAGA was used as a potent surrogate of the natural human *FSHB* promoter. On the other hand, we used a luciferase-reporter containing the human *TRHR* promoter^34^. Co-transfection of increasing concentrations of IGSF1 resulted in increased basal activity of the *TRHR* promoter, in a dose-dependent manner (Fig. 4C). Therefore, IGSF1 exerts opposite transcriptional effects over two critical gene promoters in pituitary physiology, namely activation of *TRHR* in thyrotropes and repression of *FSHB* in gonadotropes.

### The activin pathway mediates the effects of IGSF1 on the CAGA promoter, but not those on the TRHR promoter

To further investigate the effects of IGSF1 on Activin-Smad signaling, we performed a dose-response curve of Activin on the CAGA transcriptional activity in the absence/presence of fixed amount of IGSF1 (Fig. 4D). IGSF1 exerted a potent inhibitory effect, reducing responses to Activin more than six-fold (Fig. 4D). The activin effect was abolished by SB431542, an inhibitor of activin type I receptors, independently of IGSF1 (Fig. 4E). Furthermore, IGSF1 was more potent than DSmad2, a truncated protein with dominant negative effect over the wild type Smad2, which reduced CAGA activity by 40% in comparison with the 70% reduction of IGSF1 (Fig. 4E)^35^. ALK4, the type I activin receptor, is expressed in gonadotropes^36^. Transfection of a constitutively active (ca) mutant (caALK4) increased three-fold the basal transcriptional activity of CAGA in the absence of activin A (Fig. 4D). Expression of IGSF1 was also able to reduce such unstimulated caALK4-dependent transcriptional activity by 50% (Fig. 4D). These results indicate that IGSF1 is a strong repressor of the activin pathway on the CAGA promoter through a mechanism involving the ALK4 receptor. Although IGSF1 repressed the basal activity (Fig. 4A), we were unable to detect induction of this minimal human FSHB promoter by Activin in these cells (data not shown). It is known that Activin induction of the human *FSHB* gene in gonadotropes requires far upstream 5′ and downstream 3′ sequences (on exon 2 and exon 3) that were absent in our construct^31^,^37^.

As a model of pituitary gonadotrope we use the rat pituitary cell line RC-4B/C1, described as presenting a majority of cells staining for FSH and LH protein expression^38^. In those gonadotrope cells, Activin induced a strong increase in CAGA activity of near 20-fold that was reduced to 5-fold in the presence of IGSF1 (Fig. 4F). As expected^31^,^37^, we were again unable to detect induction of the minimal human *FSHB* promoter by Activin in these RC-4B/C1 gonadotrope cells, although IGSF1 repressed the basal activity (data not shown). On the other hand, we studied the intracellular protein expression of Fsh beta using western blot (Fig. 4F). Fshb was induced by Activin, and repressed when IGSF1 was transfected, confirming in a direct way the repression of Fshb protein expression by IGSF1 in gonadotropes (Fig. 4F).

### TGFβ1 pathway mediates the effects of IGSF1 over the TRHR promoter

A parallel set of experiments was performed using the human *TRHR* promoter as target of IGSF1 effects. However, neither Activin A nor SB431542 influenced the stimulatory effect of IGSF1 on the basal activity of the *TRHR* promoter (Fig. 4G). Similarly, neither the inhibitory DSmad2 nor the constitutively active ALK4 receptor (caALK4) altered IGSF1 stimulation of *TRHR* promoter (Supplemental Fig. 6). These results indicate that IGSF1 action on the human *TRHR* is independent of activin A and must be mediated by an alternative signaling cascade.

TGFβ pathways are active in multiple processes of pituitary development, differentiation and function. TGFβ1 is known to be expressed in lactotropes, gonadotropes and thyrotropes^39^. Since prolactin secretion is negatively modulated by TGFβ1 and positively regulated by TRHR^40^, we tested the hypothesis that TGFβ1 could down-regulate *TRHR* expression and, in turn, IGSF1 could counteract this effect. HEK293FT cells were transfected with the *TRHR* promoter construct and treated with different doses of TGFβ1 in the absence or presence of IGSF1 (Fig. 4H). TGFβ1, from 0.25 to 5 ng/ml, significantly reduced *TRHR* transcriptional activity but this effect was not only abolished but completely reversed in the presence of IGSF1 (Fig. 4H).

To confirm such IGSF1 effect on the endogenous *TRHR* gene, we transfected IGSF1 in rat pituitary GH4C1 cells, a somato-lactotrope cell line endogenously expressing Trhr, and measured Trhr mRNA expression by qRT-PCR (Fig. 4I). Indeed, TGFβ1 could strongly repress *Trhr* expression, an effect blocked by the inhibitor SB431542. IGSF1 was able to block TGFβ1 repression, preventing the TGFβ1-induced down-regulation of endogenous *Trhr* expression. Interestingly, TGFβ1 was also able to repress *Prl* mRNA expression, but IGSF1 did not alter such repression (Fig. 4I). These results indicate that IGSF1 action on *TRHR* is mediated by a specific alteration on the TGFβ1-Trhr signaling pathway.

TGFβ binds to TGFβRII, the complex recruits and activates TGFβRI, which initially phosphorylates SMAD2 at Serine residues in the C-terminal tail, leading to modulation of other intracellular pathways^41^. Since IGSF1 is a plasma membrane protein and co-localizes with Cadherins (Supplemental Fig. 4), we explored the immediate signaling induced by TGFβ by measuring the phosphorylation of SMAD2 C-terminal tail (p-Smad2) in pituitary GH4C1 cells. We treated with TGFβ1 for one hour in the absence or presence of IGSF1. As expected, TGFβ1 caused a potent p-Smad2 induction that was blocked by the TGFβRI inhibitor SB431542 (Fig. 4J). IGSF1 markedly reduced p-Smad2 signal by 40% with respect to total SMAD3, or by 70% with respect to total loading of protein (Tubulin) (Fig. 4J). It is well known that Smads are weak transcription factors requiring a particular context of other transcription factors to repress/activate a particular gene within a cell^42^,^43^ and this also happens in human endocrine cells^41^. We concluded that IGSF1 negatively regulates the TGFβ pathway, interfering with Smad2 activation which, in the thyrotrope intracellular context, leads to abrogation of the TGFβ effects on the *TRHR* gene.

## Discussion

IGSF1 deficiency is a recently identified human disorder of thyroid and gonadal hormone axes whose pathogenic mechanisms at the molecular level are not established.

Based on the long-term hormone follow-up of the patient in whom the disorder was originally described^3^, we here unveil two independent transcriptional effects of IGSF1 in pituitary physiology, stimulation of TRHR and repression of FSH synthesis, whose failure is mechanistically consistent with the hallmark features of human IGSF1 deficiency, central hypothyroidism and macroorchidism.

### Disruption of the thyroid hormone axis in IGSF1 deficiency

Central Congenital Hypothyroidism (CCH) is usually regarded as a mild form of hypothyroidism. However, recent surveys show that it can be clinically severe^44^. Despite this, CCH is not routinely screened for in most countries, since detection of TSH elevations is the commonly used tool to diagnose (only) thyroidal hypothyroidism in babies. Our patient showed biochemically and clinically severe hypothyroidism at birth. Despite the early diagnosis (based on clinical manifestations) and treatment of his hypothyroidism, fine motor coordination deficits are present in the patient as an adult. Attention deficiency, although mild, has been previously described in affected male patients treated from birth^45^,^46^. This suggests prenatal, non-recoverable damage of brain development by *in utero* hypothyroidism in severe cases of IGSF1 deficiency. If undetected, neonatal hypothyroidism in the degree seen in our patient would likely have resulted in psychomotor retardation^44^.

At diagnosis, free T4 was low but TSH was in the normal range, suggesting impaired TSH secretion and/or reduced TSH bioactivity. The latter was confirmed through a highly sensitive *in vitro* assay showing markedly decreased serum TSH biopotency against the TSH receptor. Together with the poor TSH response at the TRH test, this indicates that hypothyroidism in IGSF1 deficiency is a combination of quantitative and qualitative (glycosylation) defects of TSH secretion^20^,^21^.

In fact, biological potency of TSH is determined by the glycosylation pattern generated on the molecule by pituitary enzymes (glycosyltransferases) whose activity is in turn controlled by TRH–TRHR signaling in thyrotropes^21^. TSH biopotency in our patient was not only reduced but also insensitive to TRH stimulation, which is consistent with our experimental findings that the ultimate molecular mechanism for hypothyroidism in this disorder is the decrease of *TRHR* expression.

We showed that the human *TRHR* gene promoter is transcriptionally repressed by TGFβ1, an active signaling pathway previously studied in primary cultures of pituitary cells^40^. In turn, this effect is negatively modulated by IGSF1 in a specific way since other genes regulated by TGFβ1 like *Prl* were not affected. IGSF1 modulates the immediate SMAD2 phosphorylation and activation at the plasma membrane, indicating that its blocking action is mediated, at least partially, through the SMAD pathway. Since SMADs are so heavily dependent on the intracellular context^41^,^42^,^43^, a partial downregulation of SMAD2 activation could lead to abrogation of the TGFβ1 effects on the *Trhr* gene, while it is not enough to alter *Prl* repression. Another non-SMAD pathway that could be affected by IGSF1 is the Pit-1 regulation of the *TRHR* promoter^47^. Pit-1 is a major factor in thyrotropes, and there are two active biding sites in the proximal promoter of the *TRHR* gene. Future work is required to clarify whether Pit-1 regulation of TRHR is dependent on or enhanced by IGSF1.

**Figure 5:**
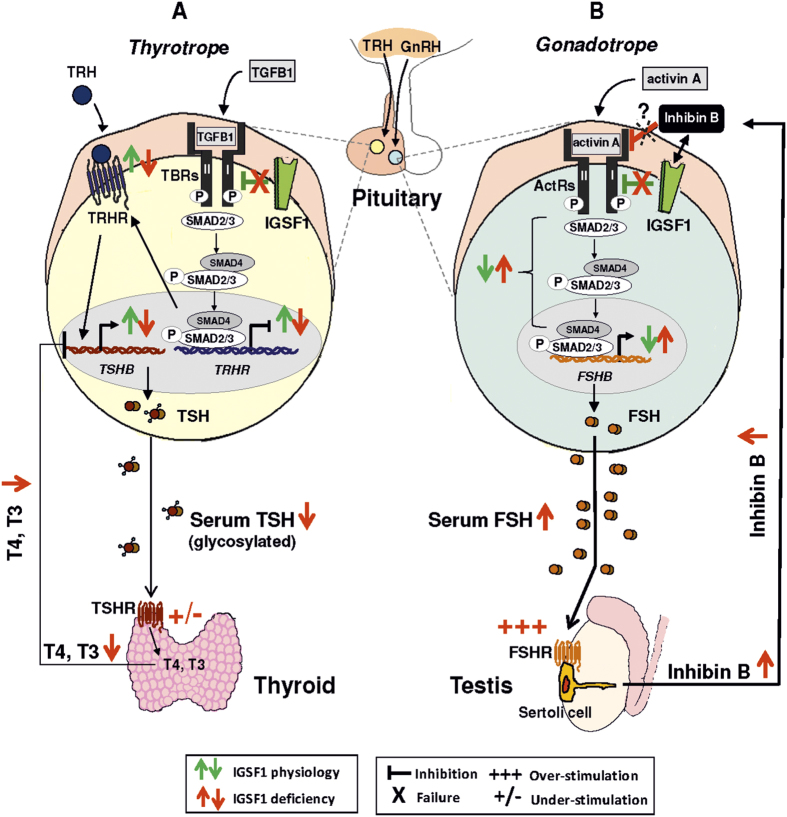
Proposed model for IGSF1 functions and molecular mechanisms involved in IGSF1 deficiency. (**A**) In thyrotropes, TGFβ1 activates TGFβ receptors (TBRs) and stimulates Smad2/3 signaling cascade leading to repression of the *TRHR* gene, which reduces the TRH-TRHR signaling over the *TSHB* expression. IGSF1 (green symbols) represses TGFβ1 pathway, positively regulating *TRHR* expression and therefore enhancing TRH-TRHR signaling, TSHB synthesis and bioactivity (glycosylation) and TSH secretion from the pituitary. In the thyroid, TSH stimulates the TSHR and the synthesis of thyroid hormones (T4, T3) which exert a negative feedback over *TSHB* expression at the pituitary. IGSF1 deficiency (red symbols) reduces TSH synthesis and bioactivity, leading to central hypothyroidism. (**B**) In gonadotropes, activin A binds activin receptors (ActRs) and stimulates Smad2/3 pathway to increase *FSHB* transcription. FSH stimulates FSHR of Sertoli cells in testis, producing inhibin B, which exerts negative feedback over pituitary *FSHB* expression. IGSF1 (green symbols) inhibits activin A pathway, negatively regulating *FSHB* expression. IGSF1 is proposed here as a mediator of inhibin B-repressive effects on *FSHB* production (black double-arrow). IGSF1 deficiency (red symbols) leads to over-secretion of pituitary FSH, inducing proliferation of Sertoli cell mass (macroorchidism) and excess synthesis of Inhibin B which, however, seems unable to exert the negative feed-back over the synthesis of FSH at the pituitary, representing a state of resistance to Inhibin B. *TRH: thyrotropin-releasing hormone, GnRH: gonadotropin-releasing hormone*, TGFβ*1: Transforming Growth Factor beta 1 protein, TBRs:* TGFβ*1 receptors, TRHR: thyrotropin-releasing hormone receptor, TSHB: thyrotropin beta subunit gene, TSHR: thyrotropin receptor, T4: thyroxine, T3: triiodothyronine, ActR: activin A receptors, FSHB: follicle-stimulating hormone beta, FSH: follicle-stimulating hormone, FSHR: follicle-stimulating hormone receptor*.

IGSF1 is capable of increasing *TRHR* expression, and its deletion in our patient is consistent with a decrease in TRHR molecules at the membrane of thyrotropes, a known model for central hypothyroidism^1^ (Fig. 5A). These results fully agree with the low Trhr mRNA levels observed in the pituitary of *Igsf1* deficient mice^4^. We identified here a mechanism whereby IGSF1 deficiency down-regulates endogenous TRHR, a pivotal molecule at the crossroad of TSH secretion signaling. Notwithstanding, Igsf1 mRNA was recently detected in the rat hypothalamus, mainly in glial cells (Gfap positive), but also in a small subset of TRH neurons^22^. Therefore, we cannot exclude other roles for IGSF1 at the hypothalamus that could also contribute to the phenotype of our patient. The intriguing possibility exists that hypothalamic Igsf1 contributes to the positive expression and pulsatility of Trh secretion and, thus, Igsf1 genetic defects could, in addition to reducing TRHR functionality in thyrotropes, decrease the hypothalamic trophic action on pituitary thyrotropes.

### Disruption of the gonadal hormone axis in IGSF1 deficiency

We present here morphological, clinical and molecular evidence strongly indicating that a pituitary disorder may be an important pathogenic mechanism leading to macroorchidism in IGSF1 deficiency.

We show that IGSF1 is strongly expressed in gonadotropes of the young adult male rat enabling the “pituitary pathogenic hypothesis” for macroorchidism. Using an IGSF1 antibody against aa 795-1090 and western blots from human and rat pituitaries and confocal microscopy of rat pituitary sections, we have found Igsf1 protein specifically expressed in gonadotropes and thyrotropes, but not somatotropes. Our results fully agree with initial reports using double immunohistochemistry and an IGSF1 antibody made in house on the presence of Igsf1 in adult rat FSH beta +ve cells^7^. In contrast, reports in adult rat pituitary^22^ and mouse embryo^4^, both using an antibody made against aa 559–575 of IGSF1 showed staining in Pit-1 +ve cells and not in gonadotropes. We can only speculate about the reasons for these differences. The main ones are the use of a different antibody (different epitopes; different sources: ours commercial, others in house produced), technique (confocal versus optic microscopy) and rodent strain (Sprague-Dawley versus Wistar rats or mouse). Our pituitary data are supported by western blot, where only two bands >140 and 50 kDa are detected in rat and human pituitary. The molecular weight of the upper band is consistent with that of the main IGSF1 protein isoform (www.uniprot.org), and the lower band may represent a processed protein from plasma membrane recycling. Nevertheless, the pituitary expression pattern we describe for Igsf1 was confirmed by detection of Igsf1 mRNA in immune-purified populations from rat pituitary, an IGSF1 antibody-free methodology. While Igsf1 mRNA was undetectable in somatotropes, we confirmed our pituitary expression pattern by detecting Igsf1 mRNA in gonadotropes and thyrotropes from rat pituitary.

We then investigated *in vitro* whether IGSF1 could regulate pituitary FSH synthesis, showing that IGSF1 decreases basal transcription of a minimal human *FSHB* promoter in a dose-dependent manner. Furthermore, IGSF1 reduces Activin induction of Fshb protein expression in cultured rat gonadotropes. Finally, we showed that IGSF1 is a potent inhibitor of the Activin-Smad pathway, a route including ALK4, a type I receptor that mediates the regulatory effects of Activin on the mouse *FSHB* promoter^36^.

Both sets of results agree with a unique clinical finding in our patient with complete IGSF1 deletion: a neonatal excess of FSH secretion at “mini-puberty”, when the gonadal axis is transiently active in babies^16^,^17^. Later in life, an aberrantly elevated FSH-secretion in response to GnRH was observed at 6 years of age, when the gonadal axis should be completely inactive. Importantly, both findings are consistent with the recent identification of increased FSH pulsatile secretion in adults with IGSF1 deficiency^48^.

Taken together, our data integrate into a model for macroorchidism in IGSF1 deficiency (Fig. 5B) based on the pituitary de-repression of *FSHB* transcription at pituitary gonadotropes and the postnatal over-secretion of FSH at mini-puberty, which may lead to premature proliferation of testicular Sertoli cells^49^, a known cause for macroorchidism in children and adults with FSH-secreting pituitary adenomas^25^,^26^,^27^. These findings are relevant since all IGSF1-deficient patients so far identified with macroorchidism were adolescents and adults^4^, which has prevented the elucidation of the precise chronology for testicular growth in this disorder. Further supports to this hypothesis are the excessive concentrations found in our patient for Inhibin and AMH, both produced by Sertoli cells. In contrast, testosterone and spermatogenesis, both dependent on Leydig-cell function , were normal. LH was not increased at any age. This clinical picture agrees with experiments in monkeys, showing that Leydig cells are absolutely dependent on LH, and that, although FSH alone could induce some Leydig cells in the interstitium, they were occasional and poorly active^50^.

Sertoli cells, the larger cell type in the testis, are the main contributors to testicular size^51^. They are known to be not fully quiescent after neonatal minipuberty, but they continue to proliferate and moderately increase testicular size during infancy^52^,^53^. Therefore, an initially increased Sertoli cell mass at the end of mini-puberty could lead to development of macroorchidism during infancy under normal FSH levels. Alternatively, elevation of pulsatile FSH secretion could also exist in IGSF1 patients during infancy, as shown in adults with IGSF1 deficiency^48^, underlying the potential to over-stimulate Sertoli cells and causing macroorchidism. Studies of FSH pulsatility require meticulous analyses with 24 h serum sampling and, so far, they have not been performed at the pediatric age^48^. Future testing of FSH pulsatility in children with the disorder may help define the early postnatal (“mini-puberty”) and/or the infantile components for the timing of Sertoli cell stimulation in the IGSF1 deficiency.

We detected IGSF1 in germinal epithelium and (less) in Leydig cells in mice and human testis, but not in Sertoli cells. This suggests that the testicular phenotype is not due to primary failure of testicular IGSF1^49^,^54^ but to overstimulation of Sertoli cells by pituitary FSH (main known stimulus for Inhibin/AMH secretion and Sertoli cell proliferation). Other researchers also found IGSF1 protein and mRNA in mouse germinal epithelium, (less) in Leydig cells and also in Sertoli cells, but only at stages VI–XIII of the maturating tubular epithelium^22^. Our human and mouse testis tissues seem to contain seminiferous tubules full of mature spermatozoids and, in those stages, our study coincides with Joustra’s in finding Sertoli cells negative for IGSF1. We only found a minimal spotty double staining within the tubules between the IGSF1 +ve germinal epithelium and the Inhibin +ve Sertoli cells. It is an open question if these are regions of intimate attachment of both cell populations, unable to be solved by confocal microscopy, or if Sertoli cells are specifically +ve por IGSF1 at those spots. More studies in human testes need to be performed to clarify the contrasting staining in Sertoli cells, including a full range of ages from pre-pubertal to adulthood. *Igsf1*^(−/−)^ knockout mice have normal FSH, are fertile and do not show macroorchidism^10^. Therefore, these mice are not useful to explain the increased testicular size in humans. If anything, the absence of macroorchidism in the mouse model suggests that testicular enlargement is not primarily due to the local inactivation of testicular Igsf1.

We considered the possible effect of hypothyroidism on the development of macroorchidism. This clinical association was known before the implementation of universal neonatal CH screening programs, in-pubertal boys with severe and long-standing hypothyroidism^55^. The mechanism was the cross-reactive stimulation of testicular FSHR by chronic and extremely high elevations of TSH in such boys from birth^55^. In rat models, hypothyroidism induces macroorchidism as a consequence of increased Sertoli cell numbers with reduced Leydig cell numbers at puberty^56^,^57^,^58^,^59^,^60^,^61^. However, two essential differences exist between the hypothyroid conditions mentioned and our patient. First, (central) hypothyroidism was congenitally present in our patient but it was readily identified and treated from birth. Second, gonadotropin levels were increased neonatally, while in hypothyroid patients (due to Hashimoto) and rat models of hypothyroidism FSH levels are strongly downregulated^56^,^57^. Therefore, a major derangement of pituitary gonadotropes (and not hypothyroidism) seems here to be a distinct factor involved in macroorchidism in the absence of IGSF1.

On the other hand, TRHR is expressed not only in the pituitary but also in other tissues, including the testis^62^,^63^. Therefore, a possible contribution of altered paracrine action of TRH on testicular TRHR in the absence of IGSF1 may deserve consideration. However, the clinical relevance of this hypothesis is questionable due to the absence of any testicular phenotype in male patients with TRHR inactivating mutations causing congenital hypothyroidism^64^,^65^.

Finally, it is striking that the strongly elevated inhibin B levels in our patient were incapable of repressing FSH secretion, identifying a state of inhibin B resistance unrecognized in humans, and suggesting a critical role of IGSF1 as mediator of the regulation of FSH by inhibin B. Inhibin B is the major regulator of testicular-pituitary function in males through the inhibition of the Activin pathway reducing FSH synthesis and release^66^. IGSF1, formerly named *InhBP (inhibin binding protein*), was proposed as a possible inhibin B receptor in affinity chromatography experiments^7^,^8^. However, this hypothesis was ruled out after ligand-receptor binding assays could not demonstrate a direct interaction between inhibin B and IGSF1^9^. In our perspective, the failure to show direct binding between inhibin B and IGSF1 does not exclude that IGSF1 could be a “pseudo-receptor” for inhibin B. This is the case of BAMBI (BMP and Activin membrane-bound inhibitor), a membrane pseudo-receptor for different TGFβ superfamily ligands that cannot bind such ligands directly, but through the presence of type II activin receptors^67^. In light of our findings, the intrinsic molecular dialogue between IGSF1 and inhibin B in pituitary gonadotropes warrants further investigation.

In summary, we revealed here two distinct molecular effects of IGSF1 consistent with the development of central hypothyroidism and testicular enlargement in IGSF1 deficiency. IGSF1 negatively modulates two TGFβ pathways: an inhibitory TGFβ1 pathway over the *TRHR* in thyrotropes and a stimulatory activin pathway over the *FSHB* in gonadotropes. Therefore, pituitaries harboring IGSF1 defects may not synthesize enough TRHR, leading to a type of central hypothyroidism combining low TSH synthesis and bioactivity. Likewise, they may not properly repress FSH secretion, causing serum FSH elevation^48^ which may lead to stimulation and proliferation of testicular Sertoli cells and macroorchidism. Thus, the major clinical features of the X-linked syndrome of IGSF1 deficiency may associate with the decreased pituitary actions of IGSF1. IGSF1 emerges as a critical regulator of different TGFβ superfamily pathways in the pituitary.

## Methods

### Hormone determinations and stimulation tests

TSH was determined with Third Generation TSH assays on both the Immulite 2000 (Siemens Healthcare Diagnostics, Ltd, Camberley, UK) and confirmed by Vitros ECi (Ortho Clinical Diagnostics, UK) and by a two-site immunoenzymometric assay using two monoclonal antibodies (ST AIA-PACK TSH; Tosoh Corporation, Tokyo, Japan) (data not shown). Total triiodothyronine (T_3_), total thyroxine (T_4_) and free thyroxine (FT_4_) in serum were measured using the Vitros ECi immunoanalyzer. FSH, LH and sex hormone-binding globulin (SHBG) were measured on the Immulite 2000 analyzer. Testosterone and estradiol levels were measured using a Coat-A-Count radioimmunoassay (Siemens Healthcare Diagnostics Ltd, Camberley, UK). α subunit was measured by an immunoradiometric assay (Immunotech SAS, France), inhibin B by Oxford Bio-Innovation ELISA (OBI-DSL Ltd., UK) and anti-müllerian hormone (AMH) by ELISA kit (Immunotech SAS, France).

For hormone assays different compounds were tested to determine specificity of the assay and none showed significant interference; cross-reactivity assays demonstrated ~100 times higher specificity for the hormone. Analytical limits of detection and dynamic range of the hormone assays are shown in Supplemental Table 1.

TRH stimulation test was performed after four weeks L-T4 treatment withdrawal. Serum TSH and prolactin were measured at −15, 0, 15, 30, 60, 90, 120, and 180 min after intravenous administration of TRH (Protirelin, 7 μg/kg body mass; max 200 μg). An inadequate TSH response was defined as a peak concentration value below 14 mIU/l and the peak/basal TSH ratio as described by van Tijn *et al*.^68^. For the study of TSH bioactivity two additional serum samples were collected at 0 and 180 min after TRH administration.

GnRH stimulation test was performed at 9 a.m. Serum FSH and LH were measured at −15, 0, 15, 30, 45 and 60 minutes after intravenous administration of GnRH (Luforan, 100 μg, Serono). Pubertal and postpubertal normal responses to GnRH were defined as described by Resende *et al*.^69^.

### Mutation screening

All coding regions of *TSHB*, the gene encoding the specific TSH beta subunit, *TRHR*, the gene encoding the TRH receptor, and *CGA*, the gene encoding the glycoprotein hormones alpha chain were amplified by PCR using appropriate primers flanking each exon. PCR products were purified and directly sequenced on an automated DNA sequencer (3100 Genetic Analyzer, Applied Biosystems).

### Comparative Genomic Hybridization Arrays (CGH Array)

High-density aCGH were performed using SurePrint G3 Human CGH Microarray 1 × 1 M (Agilent), an oligonucleotide chip that contains 963,029 distinct biological features with a probe spacing 2.1 KB overall median probe spacing (1.8 KB in Refseq genes) and Content sourced from - UCSC hg18 (NCBI Build 36).

Array experiments were performed as recommended by the manufacturer (Agilent Technologies, Santa Clara, CA, USA). 500 ng of DNA from the patient and a reference sample of the same sex (Promega, Madison, WI, USA) were double-digested using AluI and RsaI for 2 h at 37 °C. Enzymes were inactivated at 65 °C for 20 minutes and each digested sample was labeled with Cy5-dUTP by random priming at 37 °C for 2 h (Genomic DNA Enzymatic Labelling Kit Agilent). Labeled products were column-purified (Microcon Ym-30 filters, Millipore Corporation). The hybridization was performed at 65 °C with rotation for 24 h after probe denaturation and pre-annealing with Cot-1 DNA. The array was analyzed with the Agilent scanner using the Feature Extraction software (v9.1 Agilent Technologies). Comprehensive description of the statistical algorithms is available in the user’s manual provided by Agilent Technologies.

### Confirmation and determination of precise deletion size

To confirm and define the deletion size and break points, it was performed a long PCR (Expand 20Kb^PLUS^ PCR System, Roche Diagnostics; Mannheim, Germany) and Sanger sequencing on the genomic DNA of the patient and parents, following manufacturer instructions. Oligonucleotide primers were designed in the flanking regions of the deletion as defined by CGH-Array taking into account the average densities of the array both in the backbone and the targeted region. For the 207,873 bp deletion in *IGSF1* gene, primers successfully determining deletion size were: Forward: 5′-gctaaccccctgtgtagtgtg-3′ and Reverse: 5′-actctgaccctcccctcct-3′.

### *In vitro* TSH bioactivity assay

TSH bioactivity of patient’s sera was studied on thyrotropin receptor (TSHR)-transfected cells in luciferase reporter assays and compared with the bioactivity of recombinant human TSH (rhTSH). Human Embryonic Kidney (HEK293T) cells were stably transfected with a TSHR cDNA (generous gift from Dr. Lado-Abeal). Cells were cultured in DMEM/F12 with GlutaMax medium supplemented with 10% fetal calf serum (FCS), 100 IU/ml penicillin, and 100 mg/ml streptomycin and under Zeozin (Invitrogen) treatment (200 μg/mL) to maintain the TSHR expression. HEK-TSHR were transfected with 2 μg of the pcCRE(6)-luc reporter plasmid containing 6 CRE (cyclic-AMP-Responsive-Element) promoter sites, and 1 μg of the transfection efficiency *Renilla* luciferase plasmid (pRL-SV40; Promega) in T25 flask overnight using FuGene 6 (Promega) according to the manufacturer’s instructions. HEK-TSHR cells transfected with luciferase and renilla plasmids were seeded in a 96-well plate and after 8 h the growth medium was removed and stimulated cells with three different dilutions of human sera (1:2, 1:4, 1:8) and the recombinant human hormone rhTSH (Genzyme) as control in DMEM/F12 with GlutaMax medium with 0.1% BSA and pen/strep for 18 hours. After 18 h the cell-stimulating media was removed and replaced them with 25 μL of lysis buffer (25 mM Trisphosphate pH 7.8, 15% glycerol, 1% Triton X-100, 1 mM DTT and 8 mM MgCl_2_). Luciferase assays was performed using a Dual-Glo luciferase assay system as described in the manufacturer’s instructions (Promega). The firefly luciferase activity was measured adding 25 μL of luciferase reagent to lysates and analysed luminescence at a microplate luminescence counter (TopCount-Nxt tm, Packard BioScience Company). *Renilla* luciferase activity was assessed by adding an equal volume of Dual-Glo Stop & Glo substrate and measuring again in the luminometer. Firefly luciferase activities were normalized to the corresponding *Renilla* luciferase activities.

To test for unspecific responses from glycoprotein hormones in our assay, an experiment with parental HEK293 cells (without TSHR expression) transfected with pcCRE(6)-luc/pRL-SV40 was performed, showing no luciferase production in the absence of receptor (Supplemental Fig. 3) even during stimulation with the highest dose of human recombinant hormones. Further experiments showed there was no cross-reactivity in our luciferase assays between rhTSH and the FSH receptor (FSHR), nor between recombinant human (rh)FSH and the TSHR (Supplemental Fig. 3B and C).

Experiments were performed in triplicates, and repeated in three or more independent replicates. Data were represented as Relative Response Ratio (RRR), incorporating positive (pcCRE(6)-luc/pRL-SV40 transfected cells stimulated with maximal concentration of recombinant hormone) and negative control (pcCRE(6)-luc/pRL-SV40 transfected cells stimulated with the medium alone) wells within the same plate.

### Human, rat and mice tissues

Rat pituitaries were obtained from young adult (200 g, 60 days) male Wistar rats (Animal House, University of Santiago de Compostela) and immediately frozen after dissection. Neuropituitary was discarded (rAP). The area around the marginal zone (stem cell niche) was manually dissected and discarded resulting in the majority of the adenopituitary. A single tissue microarray (TMA) was prepared with the human testes using formalin-fixed paraffin-embedded (FFPE) samples from the hospital tissue bank. Mice testicles were obtained from young adult male (60 days).

### Western blot of pituitary tissue

Frozen pituitary fragments were mixed with 200 μL hot 1% SDS at 95 °C, homogenized in a polytron (Dremel, Racine, WI) during 10 sec and further incubated for 5 min at 95 °C in a thermoblocker. 1.5 vol of Triton Buffer (50 mM Hepes pH 7.5, 150 mM NaCl, 10% Glicerol, 1% Triton, 5 mM EDTA, 1.5 mMMgCl, 0.1 M PMSF, 5 mg/ml Aprotinin, 2% Na3VO4, 0.1 M NaPyrophosphate) was added and sample homogenized through a 25 g syringe followed by an incubation in ice for 20 min. Eppendorf tubes were centrifuged at 14000 rpm for 5 min at 4 °C. The supernatant was kept at −80 °C. 75 μg of proteins were loaded per lane.

### Immune Purification of secretory cell populations with magnetic beads and qRT-PCR quantification

The immune purification of secretory cell populations was established in our lab previously^70^. We have modified the protocol for RNA quantification as described^71^. Briefly, six pituitaries were dissected and dispersed to single cells with collagenase and DNAse for 20 minutes and washed three times with DMEM. Single-cell dispersions were fixed by adding 1 vol ethanol 100% (final concentration: 50%) for 15 minutes in ice. Cells were washed three times with PBS, divided into five tubes, three for incubation with the respective anti-hormone antibody (see Supplemental Table 2), one for non-antibody control beads purification and one for direct extraction as control for single-cell dispersion. Tubes were kept for 30 minutes in ice. After washing with MACS buffer (Miltenyi), cells were incubated with 1:4 magnetic beads coupled to anti-rabbit antibody (MACS, Miltenyi) for 15 minutes in ice. Cells were washed again once with MACS buffer and added to the column attached to the magnetic separator (Miltenyi).

RNA was extracted with Trizol, resuspended in 12 microliters of water and incubated with 1unit of RNAse-free DNAse (Thermo) for 30 min at 37 °C, followed by inactivation. Reverse-transcription was performed in 30 μl using 250 units of MMLV (Invitrogen). Quantitative PCR was performed with 1 μl of the reaction with SybrGreen (Brilliant III Ultra-fast, Agilent) in an Abyprism 7500 (Applied Biosystems) using the following primers: rattus-Gh-F-TGGCTGCTGACACCTACAAAGAG; rattus-Gh-R-CCTGGGCATTCTGAATGGAA rattus-Fshb-F-GATAGCCAACTGCACAGGACATAG; rattus-Fshb-R-ATGCAAAGCTGGATCGACTTC; rattus-Tshb-F-TCTGCGCTGGGTATTGTATGAC; rattus-Tshb-R-CAGACATCCTGAGAGAGTGCGTACT; rattus-Igsf1 (primers designed on the epitope recognized by the commercial antibody used in this work)-F-CAGCCTAGCAACACTCTGGAACT; rattus Igsf1-R-TGGACTAACAGGCTGGGCTTA; rattus-18s-F-5′CGGCTACCACATCCAAGGAA3′; rattus-18s-R-5′GCTGGAATTACCGCGGCT3′. For quantitative mRNA expression, relative ΔCt values for every gene were referred to its Tbp ΔCt in every sample, and the average of the ΔCt signal in the control unpurified cell dispersion was taken as 1 (ΔΔCt) for every gene. Experiments were performed in quadruplicate.

### Inmunohistochemistry of testicular tissue

Immunohistochemical studies were performed in paraffin section of adult human testes with anti-IGSF1 rabbit polyclonal antibody (GeneTex) and different testicular antibodies (Supplemental Table 2). Sections were stained using an automated system DAKO Austostainer. Antigens were retrieved with the Dako buffer citrate pH 6 in a DAKO PT Link for IGSF1 and pH 9 for inhibin for 60 min. Immunohistochemistry was performed using the Dako Envision Flex kit. Immune reactions were developed with diaminobenzidine, and sections were counterstained with Harris’ hematoxylin. As negative controls, adjacent sections were subjected to the same immunohistochemical procedure omitting the primary antibody.

### Immunofluorescence and confocal co-localization

Tissues were fixed in neutral formalin and included in paraffin as described^70^,^72^. 3 μm sections were mounted in superfrost coated slides (Thermo) and directly processed for automatic antigen retrieval in a PT-link (DAKO) using the pre-programmed cycle at 97 °C followed by blocking in 50 mM NH4Cl during 20 minutes. Antibody incubations (Supplemental Table 2) were intermixed with washing in PBS. Finally samples were mounted in Fluorogel (Electron Microscopy) with 2 μg/ml DAPI (Sigma). Negative controls were performed in the absence of both primary antibodies, or with one primary but not the other, always followed by both secondary antibodies and DAPI. Microscopy analysis was performed in a TCS SP5 X white light laser including a UV laser configuration (Leica) by exciting at 405, 492 and 552 nm while capturing maximal emission at 414–479, 498–547 and 565–624 nm sequentially between frames. Whole Sections were recorded first at low magnification (20x) in three pictures (right lobe, centre and left lobe); thereafter at least 6–10 fields per area were recorded at 63x followed by zooming in (z4) for some specific cell groups. Negative controls without 1^st^ antibody did not show any intense signal able to be recorded at 498–547 and 565–624, but presented intense staining at 419–479 (DAPI); negative control omitting IGSF1 antibody presented a strong signal for hormone (Supplemental Fig. 7).

Quantifications were performed using ImageJ/Fiji (Rasband, W.S., Image J, U. S. National Institutes of Health, Bethesda, Maryland, USA, http://imagej.nih.gov/ij/, 1997–2012). Around 400–500 cells were counted per picture in at least six independent pictures from three different pituitaries.

### In silico characterization of TRHR and FSHB promoters

To identify potential Smad binding elements (SBEs) in the *TRHR* and *FSHB* promoters, the Multi-genome Analysis of Positions and Patterns of Elements of Regulation (MAPPER2, http://genome.ufl.edu/mapper/mapper-main) program was used. MAPPER2 is a platform for the computational identification of transcription factor binding sites (TFBSs) in multiple genomes that combines the use of TRANSFAC and JASPAR databases. The platform and databases include Smad3 (TAGCAGACAG) and Smad4 (GTGGGGCAGCCACT) consensus sequences.

### Expression plasmids, reporter constructs and reagents

The expression vector for human pCMV-SPORT6.1-IGSF1 was purchased from Cultek. Human pGL2-pTRHR luciferase reporter vector constructed from −2530 to +1 (initiator ATG position) has been described in Matre *et al*.^34^. The human *FSHB* promoter was PCR-amplified in a 995 bp product comprising the sequence −875 to +120 relative to the transcription start site (NM_000510) using 5′-gctgttgtccttttgctccagtc-3′ as forward primer and 5′-gctctagttttgtcctccatgtcc-3′ as reverse primer, and cloned into the pGL3 basic vector (Promega). Artificial pGL3-pCAGA luciferase reporter vector containing Smad3 binding elements was provided by Dr. Jenny Visser (Erasmus MC, Rotterdam, The Netherlands) as was constitutive active pCDNA3.1-ALK4 type I activin receptor-like kinase (caALK4). Dominant-negative mutant of Smad2 into pCDNA 3.1 (DSmad2) was provided by Dr. Carmelo Bernabéu (Biological Research Center, CIB, Madrid, Spain).

Recombinant activin A (R&D Systems, Minneapolis, MN) was dissolved in PBS/0.1% BSA and used at 2–80 ng/ml. SB431542, TGFβ pathways inhibitor (Selleck Chemicals), was dissolved at 10 mM in DMSO and used at 5 μM/ml final concentration. TGFβ1 (Calbiochem, Merck-Millipore) was dissolved in 4 mM HCl-0.1%BSA and used at a range of concentrations 0.125–5 ng/ml. When not indicated, it was used at 2.5 ng/ml.

### Transient transfections and luciferase assays

HEK293 cells were cultured in DMEM supplemented with 10% fetal bovine serum (FBS, Gibco), 100 IU/ml penicillin, and 100 mg/ml streptomycin at 37 °C in 5% CO_2_. One day before transfection, cells were seeded in 12-well culture plate. At 50% confluency, HEK293FT cells were transfected with FuGene 6 transfection reagent (Promega) using 500ng of DNA per well or increasing concentrations of IGSF1 plasmid (0, 50, 100, 150, 200 ng/well) and a reporter vector in constant doses (300 ng/well), for dose-response curve experiments; and with 250ng total DNA per well with ratio 1:3 between the expression plasmid and the reporter vector, for activin pathway experiments, or with kit V-program A023 (Amaxa) using 1.25 mg total DNA per cuvette distributed in 0.5 mg human pTRHR-luc or pCAGA-luc, 0.5 mg IGSF1 or Empty-CMV, and 0.5 mg pCMV-bGal. 24 hours after transfection, cells were treated with recombinant protein activin A with/without SB431542 or vehicle treatment in DMEM with 0.2% FBS and pen/strep for 24 hours.

To test TGFβ pathway, HEK293FT cells were transfected with 500 ng of total DNA per well: 200 ng of IGSF1 plasmid or pcDNA and 300 ng of pTRHR reporter vector. 24 hours after transfection, cells were treated with recombinant TGFβ1 with/without SB431542 or vehicle treatment in DMEM with 0.2% FBS and pen/strep for 24 hours. In all cases co-transfection with CMV-renilla or CMV-β-gal reporter plasmid was performed. Dual-luciferase reporter assay (Promega) was accomplished as described in the manufacturer’s instructions (Promega), and measuring using a luminometer Glomax 96 microplate (Promega). Experiments were performed in quadriplicates, and repeated in three or more independent replicates.

### Transient transfection, real-time quantitative PCR and western blot in pituitary cells

GH4C1 cells were cultured as described previously^72^. Three days later, cell were transfected with 3 μg of IGSF1 or empty vector (pcDNA3) plasmid/well in 12-well culture plates. Transfection was performed by Nuclefection (Amaxa, Lonza) using the A020 program as described^70^,^72^. 30 hours after transfection, cells were treated with TGFβ1 for 24 hours. RC-4B/C1 were cultured as described^38^ except that DMEM was 1gr/l glucose. They were transfected with 0.5 μg DNA/well Turbofect (Thermofisher) for 24 hours.

RNA was extracted with Trizol. 1 μg of total RNA was incubated with 1unit of RNAse-free DNAse (Thermo) and reverse-transcribed as above. Quantitative PCR was performed with 1 μl of the reaction using the following primers: Trhr-F-cagatgtttcaacagcaccg; rattus-Trhr-R-ggttgtaaatcaccgggttg; rattus-Tbp-F-cttcgtgccagaaatgctgaa; rattus-Tbp-R-cagttgttcgtggctctcttattctc. For quantitative mRNA expression studies, relative ΔCt values for Trhr were referred to its Tbp ΔCt in every sample, and the average of empty vector/vehicle-treated controls was taken as 1 (ΔΔCt) for all groups. Experiments were performed in triplicates and independently repeated three times.

Proteins were extracted and phospho-SMAD2/SMAD2/3 and total Smad signaling and was performed as described^41^ and explained in Supplemental Table 2, where conditions for Fshb are included.

### Statistical analysis

Statistical significance was defined as *P < 0*.*05* and was determined by two-tailed *t* test for parametric samples after assessing that samples did follow a normal distribution with the two-samples Kolmogorov-Smirnov test (Graphpad Prism v5.0).

### Ethic statement for human studies

Human pituitary sample extracts (frozen material) and normal human testes were obtained from the Biobank of the Department of Pathology, Santiago de Compostela University Hospital (Spain), registered as GAL, n° 52 within the approved Biobank Network of the Spanish Ministry of Health (ISCIII, http://www.redbiobancos.es/Pages/Docs/triptico_donantes_A5_cerrado_141031.pdf).

All experiments were performed in accordance with relevant guidelines and regulations. All experimental protocols were approved by the Biobank Committee of the Santiago de Compostela University Hospital (Spain). Written informed consent was obtained from all subjects previous to surgery.

### Ethic statement for animal experiments

Animal experiments were performed in accordance to the license 15010/14/005 granted to CV Alvarez. All experimental protocols were approved by the official licensing Committee (“Xefatura Territorial da Conselleria do Medio Rural e do Mar”) of the Regional Government of Galicia (Spain). This Committee guarantees that animal experimentation is performed attending the ethic guidelines required by European and National legislation (RD53/2013).

### Informed consent

Informed consent for genetic studies was obtained from the index patient and his parents, according to protocols followed at the Ramón y Cajal University Hospital (Madrid, Spain), where the patient was clinically followed.

## Additional Information

**How to cite this article**: García, M. *et al*. The syndrome of central hypothyroidism and macroorchidism: IGSF1 controls *TRHR* and *FSHB* expression by differential modulation of pituitary TGFβ and Activin pathways. *Sci. Rep.* **7**, 42937; doi: 10.1038/srep42937 (2017).

**Publisher's note:** Springer Nature remains neutral with regard to jurisdictional claims in published maps and institutional affiliations.

## Supplementary Material

Supplemental Information

## Figures and Tables

**Table 1:** Long-term follow up of thyroid and gonadal hormone profiles of the patient with IGSF1 deletion.

Age	*14 d	1 mo	5 mo	^#^3y	^#^6y	8 y	9 y	10 y	11 y	12 y	12.5y	14 y
TSH *mIU*/*L*	1.4	*0.11*	*0.09*	1.07	1.52	*<0.01*	*0.08*	*<0.01*	*<0.01*	*<0.01*	*<0.01*	*<0.01*
FT4 *pmol*/*L*	*7.2*	26	23.5	*9.9*	*7.7*	16.1	11.6	14.8	11.2	*8.4*	13.5	11.7
L-T4 dose *μg*/*Kg*/*day*	0	10	5	0	0	1.9	2	1.65	1.4	1.35	1.9	1.4
FSH *IU*/*L*	**6.3**	**4.5**	1.2	0.43	0.66	0.74	0.88	1.10	0.82	1.11	1.76	2.56
LH *IU*/*L*	*0.15*	na	0.54	<0.1	0.2	0.10	0.14	0.18	0.10	0.27	0.85	0.41
Testosterone *nmol*/*L*	1	na	1	na	<0.5	<0.5	<0.5	<0.5	<0.5	0.6	13.50	11.80
^†^Testicular vol. *ml (p98 upon age*)	Na	2 (*2.8*)	2 (*2.8*)	**3** (*2.8*)	**4** (*3.5*)	**5** (*3.8*)	**5** (*4*)	**6** (*5*)	**8** (*8*)	**12** (*15*)	**20** (*24*)	**40** (*28*)

Hormone parameters are presented in chronological order along patient’s lifespan. In italics and bold, hormone values below and above normal ranges for age, respectively. *Age at clinical diagnosis of the patient. ^#^Age after 1 month levo-thyroxine withdrawal to perform two separate TRH tests. ^†^Testicular volume was measured by orchidometer until size exceeded 25 ml, when a caliper and the Lamber’s formula were used^18^.

TSH: thyrotropin, FT4: free thyroxine, L-T4: levothyroxine treatment, FSH: follicle-stimulating hormone, LH: luteinizing hormone, vol.: volume. d: days; mo: months; y: years; na: not available. Normal ranges for TSH and FT4 are from the Laboratory of the Institution where determinations were performed (TSH: 0.4–4.3 mIU/L, FT4: 11–25 pmol/L), age-related normal ranges for gonadal hormones are from refs 73, 74, 75, and for testicular volume are from ref. 52.

**Table 2:** Detailed thyroid and gonadal hormones profiles of the IGSF1-deficient patient at 14 years of age and his parents.

Age	Patient			Mother^1^	Father	Normal ranges		
	A	B	C			Male	Male	Female
	14 y	14 y	14 y	50 y	49 y	T-IV	Adult	Menopause
TSH *mIU*/*L*	*0.002*	1.7	2.0	*0.002*	2.17	*0.4*–*4.3*		
FT4 *pmol*/*L*	17.2	*8.3*	*9.1*	**26.6**	17.1	*11*–*25*		
T4 *nmol*/*L*	67	*41.4*	*40.2*	109.0	95.1	*58*–*128*		
T3 *nmol*/*L*	1.57	1.37	1.40	1.82	1.58	*1.4*–*2.5*		
L-T4 dose *μg*/*Kg*/*day*	1.4	0	0	1	0			
FSH *IU*/*L*	2.9	4.0	4.1	123.0	3.8	*0.3*–*8*	*1.5*–*14*	*35*–*150*
LH *IU*/*L*	0.6	1.0	1.1	32.8	3.4	*0.5*–*5*	*1.5*–*8*	*15*–*90*
SHBG *nmol*/*L*	20.3	11.7	11.7	78.8	31.7	*13*–*88*	*10*–*70*	*20*–*120*
Testosterone *nmol*/*L*	11.3	15.3	11.0	*0.1*	11.7	*9*–*19*	*10*–*30*	*0.5*–*3*
E2 *pmol*/*L*	97	112	85	27	104	*17*–*178*	*50*–*200*	<*50*
Inhibin B *ng*/*L*	**505**	**423**	**500**	20	236	*107*–*310*	*150*–*400*	*10*–*200*
AMH *μg*/*L*	**44.4**	**48.0**	**40.7**	0.1	**15.3**	*2*–15.5*	*1.8*–13.7*	<*0.1*

Three hormone profiles of the patient correspond to three subsequent time points: A, Under L-T4 treatment, B, Four weeks after levo-thyroxine withdrawal for the performance of a TRH test, and C, 3 hours after TRH stimulation. In italic and bold, hormone values below and above normal ranges, respectively. ^1^The mother started levo-thyroxine treatment for central hypothyroidism 1 year after diagnosis of her child with hypothyroidism. Therefore, her hormone determinations were performed under L-T4 treatment.

TSH: thyrotropin, FT4: free thyroxine, T4: total thyroxine, T3: total triiodothyronine, L-T4: levo-thyroxine treatment, FSH: follicle-stimulating hormone, LH: luteinizing hormone, SHBG: sex hormone-binding globulin, E2: estradiol, AMH: anti-müllerian hormone, y: years, T-IV: Tanner IV. Normal ranges for thyroid hormone axis and for adult male and postmenopausal female gonadal hormones are from the Laboratory of the Institution where determinations were performed. Normal ranges for gonadal hormones of a male in the Tanner IV stage are from refs 73, 74, 75, 76. *Normal range for male anti-müllerian hormone is from ref. 77.
