# Supplementary material for: The syndrome of central hypothyroidism and macroorchidism: IGSF1 controls TRHR and FSHB expression by differential modulation of pituitary TGFβ and Activin pathways
Source: Sci Rep. 2017 Mar 6;7:42937. doi: 10.1038/srep42937 (PMC5338029; doi:10.1038/srep42937)
Supplement: Supplemental Information [file srep42937-s1.pdf]

SUPPLEMENTAL INFORMATION

The syndrome of central hypothyroidism and macroorchidism: IGSF1 controls *TRHR* and *FSHB* expression by differential modulation of pituitary TGFβ and Activin pathways

Marta García [1], Raquel Barrio [2], Montserrat García-Lavandeira [3], Angela R. Garcia-Rendueles [3], Adela Escudero [1], Esther Díaz-Rodríguez [3], Darya Gorbenko Del Blanco [4], Ana Fernández [1], Yolanda B de Rijke [5], Elena Vallespín [6], Julián Nevado [6], Pablo Lapunzina [6], Vilborg Matre [7], Patricia M. Hinkle [8], Anita C.S. Hokken-Koelega [4], María P. de Miguel [9], José Manuel Cameselle-Teijeiro [10], Manuel Nistal [11], Clara V. Alvarez [3*], José C. Moreno [1*].

[1] *Thyroid Molecular Laboratory, Institute for Medical and Molecular Genetics (INGEMM), La Paz University Hospital, Autonomous University of Madrid, Spain.*
[2] *Pediatric Endocrinology and Diabetes, Ramón y Cajal University Hospital, Madrid, Spain.*
[3] *Centro de Investigación en Medicina Molecular y Enfermedades Crónicas (CIMUS), University of Santiago de Compostela, Instituto de Investigación Sanitaria (IDIS), Santiago de Compostela, Spain.*
[4] *Department of Pediatrics, subdivision of Endocrinology, Erasmus Medical Center, Rotterdam, The Netherlands.*
[5] *Department of Clinical Chemistry, Erasmus Medical Center, Rotterdam, The Netherlands.*
[6] *Functional and Structural Genomics, Institute for Medical and Molecular Genetics (INGEMM), La Paz University Hospital, Madrid, Spain.*
[7] *Department of Biosciences, University of Oslo, Oslo, Norway.*
[8] *Department of Pharmacology and Physiology, University of Rochester Medical Center Rochester, NY, USA.*
[9] *Cell Engineering Laboratory, La Paz Hospital Research Institute IdiPAZ, Madrid, Spain*
[10] *Department of Anatomic Pathology, Medical Faculty, University of Santiago de Compostela, Clinical University Hospital, Santiago de Compostela, Spain.*
[11] *Department of Anatomy, Histology and Neuroscience, Autonomous University of Madrid, Madrid, Spain.*

[*] Corresponding authors:

José C. Moreno, MD, PhD
Thyroid Molecular Laboratory. Institute for Medical and Molecular Genetics (INGEMM)
La Paz University Hospital, Paseo de la Castellana, 261
ES–28046 Madrid (Spain)
E-mail: josecarlos.moreno@salud.madrid.org
Tel: +34 912071010 (ext. 224)

Clara V. Alvarez, MD, PhD
Neoplasia & Endocrine Differentiation (P0L5)
Centro de Investigación en Medicina Molecular y Enfermedades Crónicas (CIMUS)
University of Santiago de Compostela (Spain)
E-mail: clara.alvarez@usc.es

A

1ª basal GH	2ª basal GH
11,5 ng/ml	10,2 ng/ml
Normal > 10 ng/ml	

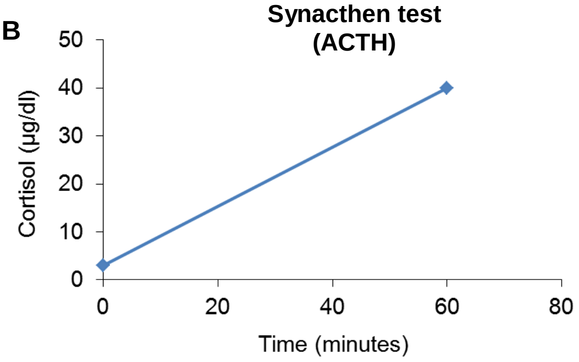

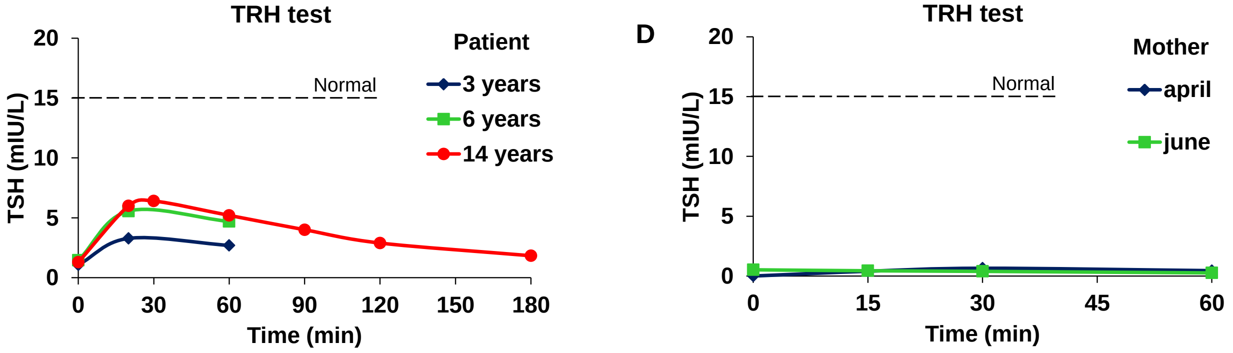

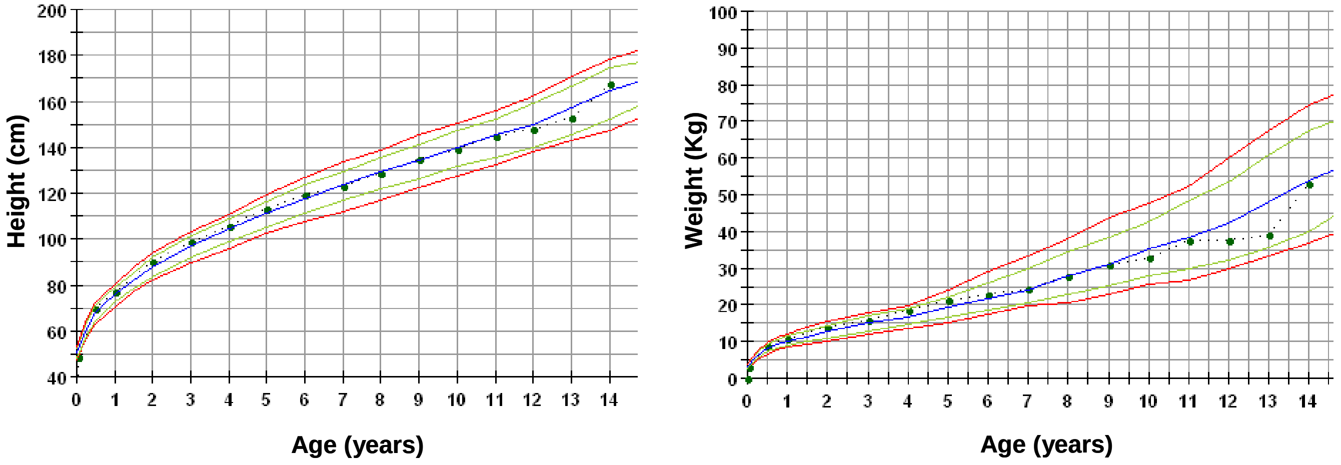

Supplemental Figure 1: Pituitary response at different stimulation tests and growth charts of the patient with IGSF1 deletion. A) At initial diagnosis of the patient, and to rule out a possible combined pituitary hormone deficiency (CPHD), serum basal growth hormone (GH) was determined in two occasions showing normal values. **B)** a Synacthen ® (ACTH) stimulation test was performed as neonate (15 days of life) with normal results (peak >20 µg/dl), discarding cortisol deficiency. **C)** TRH stimulation tests at 3, 6 and 14 years of age showed a mild improvement in the TSH peak response to TRH along with age. A similar improvement has been described in one patient with congenital central hypothyroidism (15) and could be related to the repeated exposure of the pituitary to TRH at previous tests (Beck-Peccoz P, Amr S, Menezes-Ferreira MM, Faglia G, Weintraub BD. Decreased receptor binding of biologically inactive thyrotropin in central hypothyroidism. Effect of treatment with thyrotropin-releasing hormone. N Engl J Med. 1985; 312(17):1085- 90). **D)** Repeated (two) TRH stimulation tests in the mother of the patient at 36 years of age, showed a flat TSH response to TRH, consistent with a pituitary defect not related to L-T4 treatment. **E-F)** Height and weight charts of the patient showing normal growth within the 50th percentile in both curves, further ruling out GH deficiency. A physiological and transient deceleration of height and weight is observed during peri-pubertal stages. Adult height at 20 years of age reached 175 cm, which is actually higher than his calculated target-height of 169 cm.

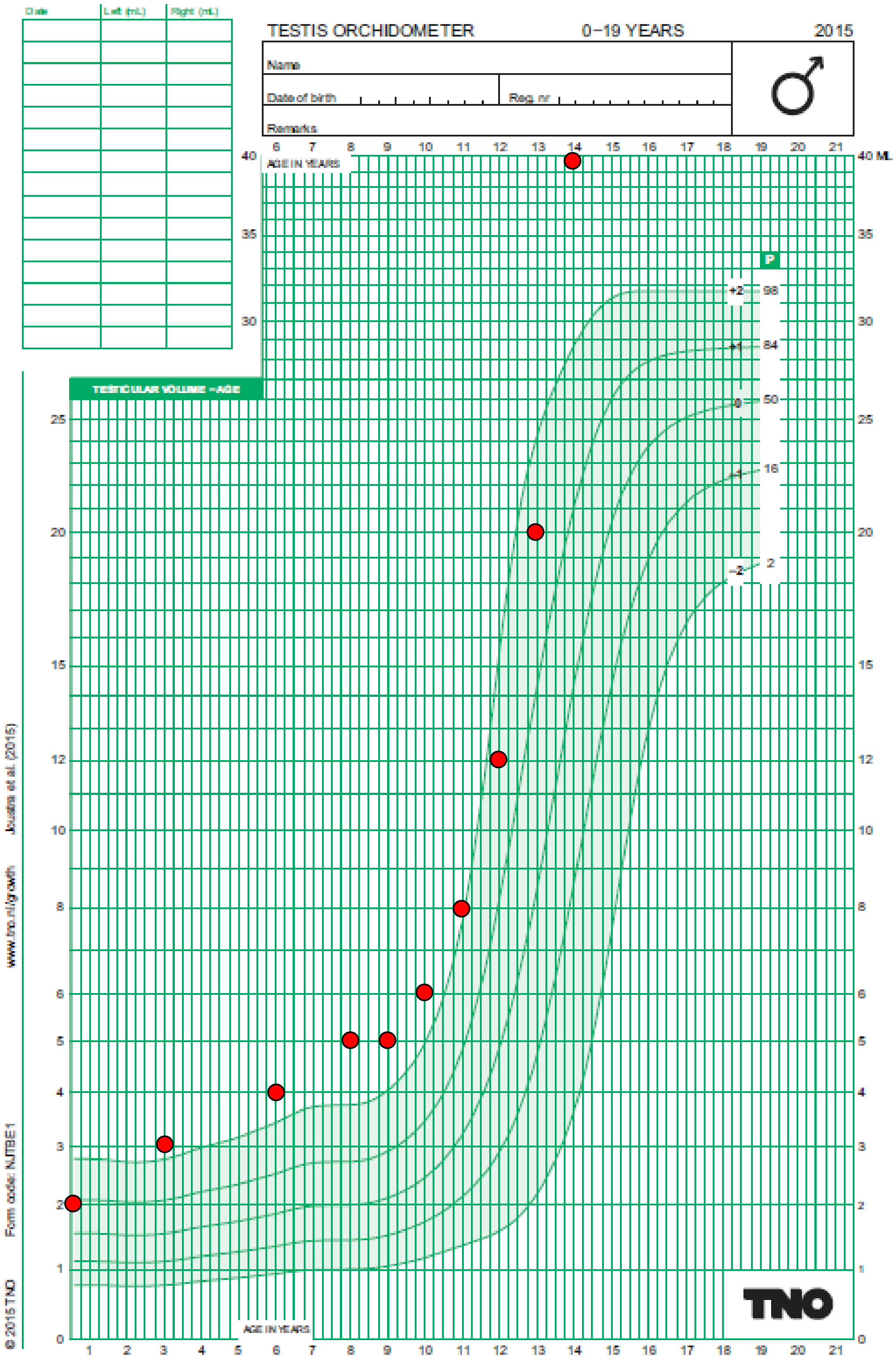

Supplemental Figure 2: Long-term follow up of patient's testicular size. Patient with complete IGSF1 deletion developed a progressive macroorchidism observed since 3 years of age, with a testicular size of 3 ml (>p98), which continued increasing above percentile 98 until 12 years of age. At the age of 14 the testicular volume markedly increased, along with the expected and normal elevation of FSH levels during puberty. Reference chart for testicular volume from Joustra SD, van der Plas EM, Goede J, Oostdijk W, Delemarre-van de Waal HA, Hack WW, van Buuren S, Wit JM. New reference charts for testicular volume in Dutch children and adolescents allow the calculation of standard deviation scores. Acta Paediatr. 2015 104(6):e271-278.

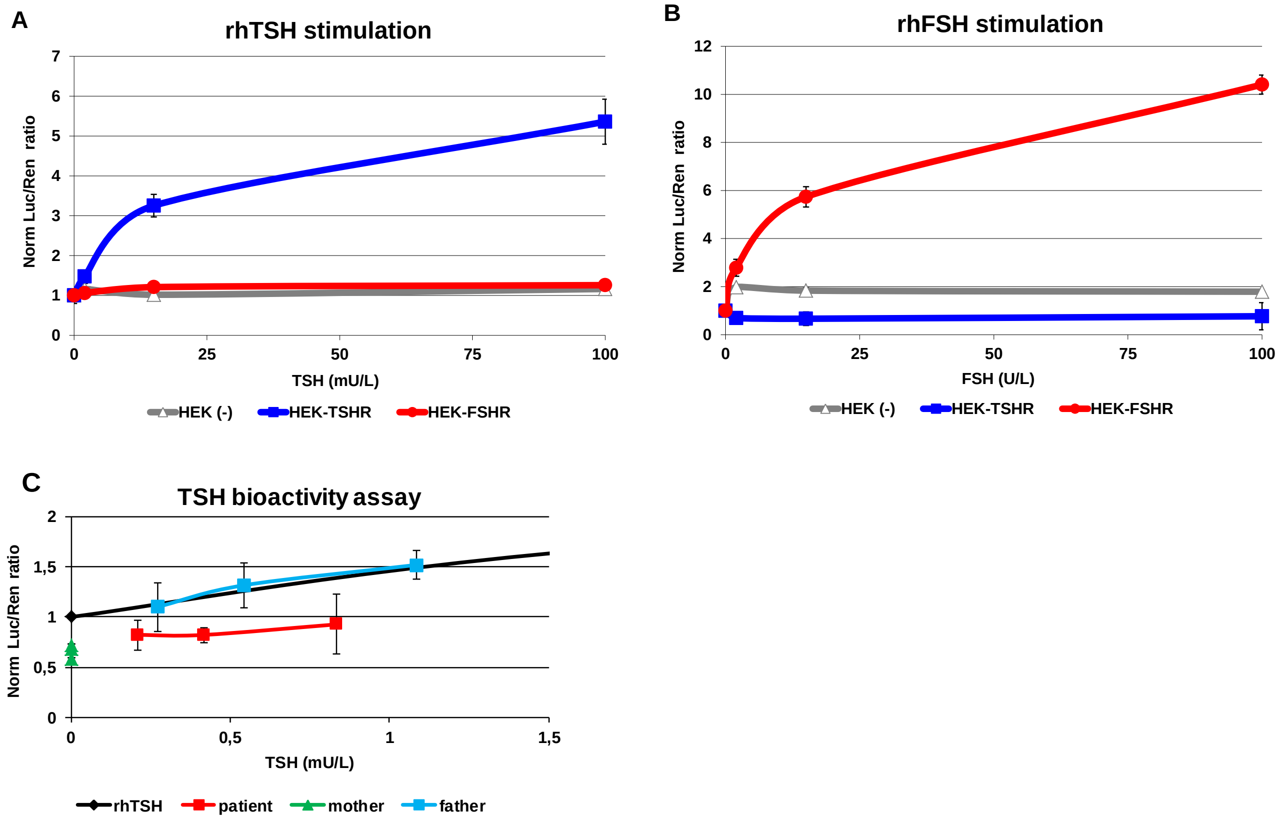

Supplemental Figure 3: Glycoprotein hormone cross-reactivity test in TSH- and FSH-bioactivity assays and TSH bioactivity of patient with IGSF1 deletion. Bioactivity of the recombinant TSH **(A)** and FSH **(B)** in untransfected HEK293 cells (grey lines) and cells transfected with either TSHR cDNA (blue lines) or FSHR cDNA (red lines). Stimulation of the TSHR by recombinant human TSH (rhTSH) is specific, without any cross-reactive stimulation of the FSHR **(A)**. Similarly, stimulation of the FSHR by recombinant human FSH (rhFSH) is also specific, without detectable cross-reactive stimulation of the TSHR **(B)**. TSH bioactivity of the sera from the mother and father as compared to the patient, showed a decreased bioactivity in patient and his mother (heterozygous carrier of IGSF1 deletion), whereas the father (without IGSF1 deletion) presented normal TSH bioactivity (C). Three different dilutions of the human sera are shown per condition (1:2, 1:4, 1:8). expressed as normalized firefly luciferase activities to the corresponding renilla luciferase activities (means ±S.E.M.).

A

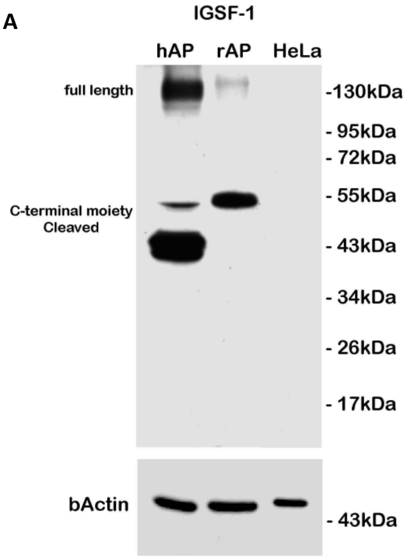

B

CORONAL SECTIONS RAT ADENOPITUITARY	
MARKER	% OF IGFS1 CELLS (RED) CONTAINING THE HORMONE MARKER (GREEN)
TSH	AP middle: 90.3% ± 4.8<br>middle towards the border with Lobes: 63.97% ± 3.6
FSH	AP Lobes: 92.33 ± 7.19 %
LH	AP Lobes: 95.11 ± 7.75 %
aGSU	99.8 ± 0.44 %
GH	0.025 ± 0.05 %
PRL	0.022 ± 0.04 %
ACTH	0.025 ± 0.05 %

MARKER	% OF SECRETORY CELLS (GREEN) CONTAINING THE MARKER (RED)						
	FSH	LH	TSH	aGSU	GH	PRL	ACTH
IGFS1	99.8 ± 0.44 %	99.8 ± 0.44 %	99.8 ± 0.44 %	99.8 ± 0.44 %	0.025 ± 0.05 %	0.022 ± 0.04 %	0.025 ± 0.05 %

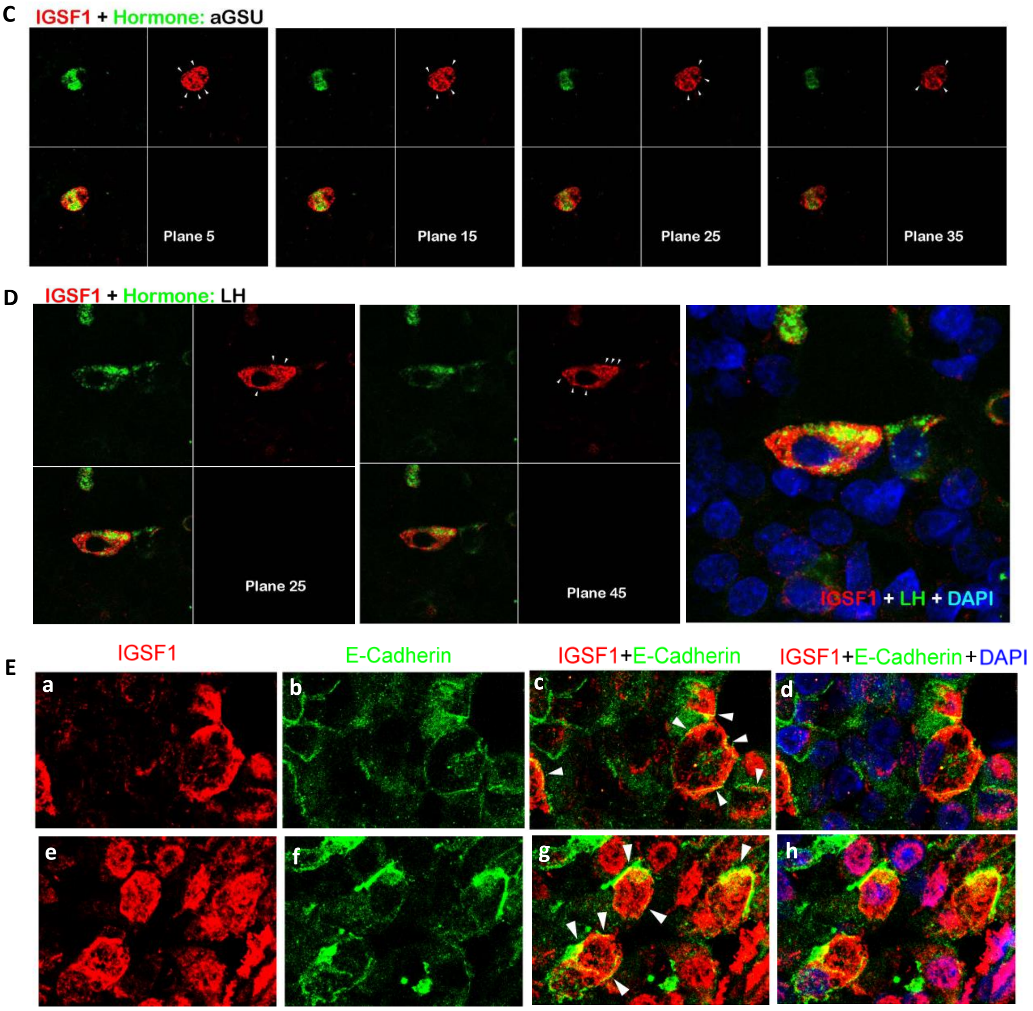

Supplemental Figure 4. IGSF1 is a pituitary protein characteristic of thyrotropes and gonadotropes residing in the plasma membrane. A) Western blot of human and rat adenopituitary (AP) using an antibody raised against the N-terminal moiety. The human HeLa cell line has been used as a human negative control. Full length IGSF1 is a protein >145 kDa. Cleaved IGSF1 C-terminal half (~55 kDa) is further processed in humans (~45 kDa). **B)** Quantification of the confocal studies co-localizing IGSF1 and the pituitary hormones. Data have been expressed in either or two ways: taking IGSF1 as the principal marker and evaluating the percentage of cells co-staining for a given hormone (above); or quantitating how many endocrine cells of a given type co-expressed IGSF1 (below). **C-D)** 4x zooming of isolated co-stained cells and IGSF1 subcellular localization. White arrowheads show plasma membrane reinforcement of IGSF1 staining at the periphery of the hormone staining through the different planes, in an aGSU+ve cell (C) and in a LH+ve cell (D). Planes were taken every 70 nm. **E)** Confirmation of IGSF1 subcellular localization through co-localization of IGSF1 (***a, e***, red) and E-Cadherin (***b, f,*** green), a plasma membrane marker, clearly showing yellow co-staining at the cell membrane (***c, g,*** white arrowheads,). Co-localization of IGSF1 and E-Cadherin plus DAPI-stained cell nuclei (***d, h***).

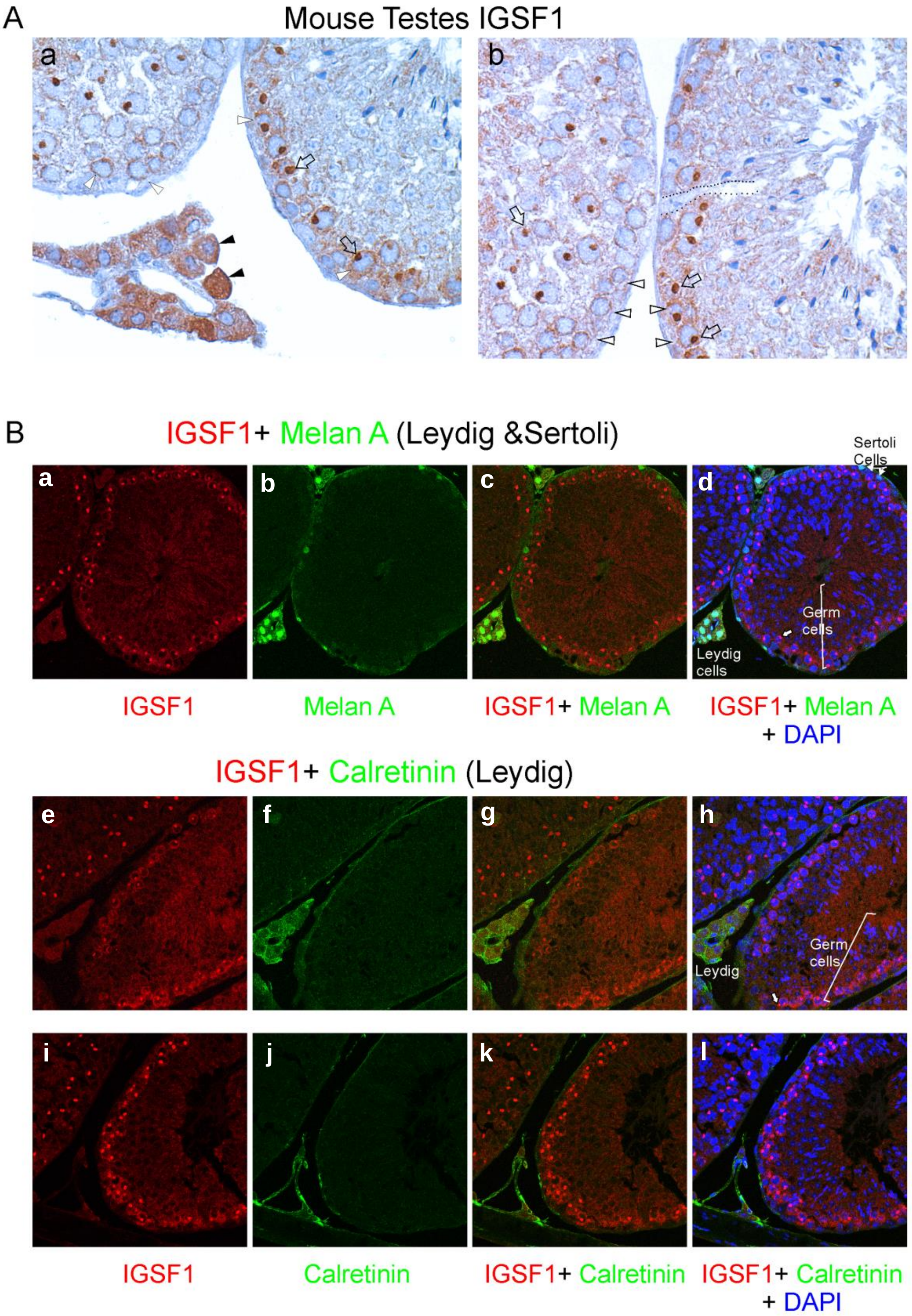

Supplemental Figure 5. IGSF1 staining of adult mouse testes reveals strong staining in cell granules that is not seen in human stainings. A) Immunohistochemistry of Igsf1 shows cells at the interstitial space (***a,*** black arrowheads) but also within seminiferous tubules, with reinforced staining in the basal layer (***a, b,*** white arrowheads). Sertoli cells seems not stained by Igsf1, as illustrated by the dotted-lined highlighted cell with its characteristic prominent nucleoli (***b***). Igsf1 stains peri-nuclear granules of the germ epithelium (thick open arrows), predominantly at the spermatogonia (***a, b***). **B)** Immunofluorescent colocalization of Igsf1 with Melan A (Leydig and Sertoli cell marker) and Calretinin (specific Leydig marker). Igsf1 (***a, e, i,*** red) is present within seminiferous tubules but also at the interstitial space. As expected, Melan A stains Leydig and some Sertoli cells (***b***, green), but does not co-localize with Igsf1 within seminiferous tubules (***c, d***). Calretinin-Igsf1 double fluorescence confirms the presence of both proteins only in Leydig cells (***g, h, k, l)***. Within tubules, Igsf1 staining is reinforced at the basal layer with marked granules, also detected by immunohistochemistry, but staining is present all-through the germ cell epithelium width (***g, h, k, l***).

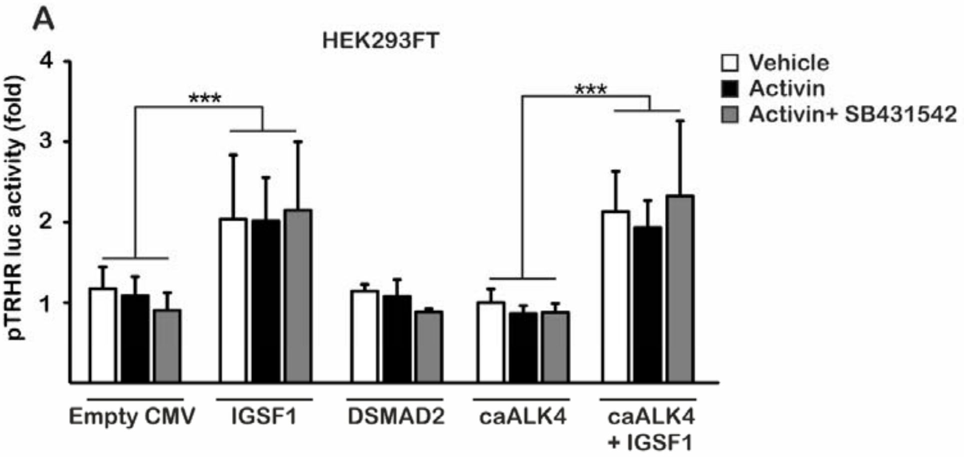

Supplemental Figure 6. Activin does not alter the expression of the TRHR promoter nor affect the postive effect of IGSF1. Transfection of IGSF1 duplicate the activity of the human TRHR promoter. Addition of Activin does not affect the basal activity nor the IGSF1 induction, and consequently the receptor inhibitor SB431542 has no effect. The promoter is affected neither by a SMAD2 dominant negative (DSMAD2) nor by an Activin receptor constitutively activated mutant (caALK4).

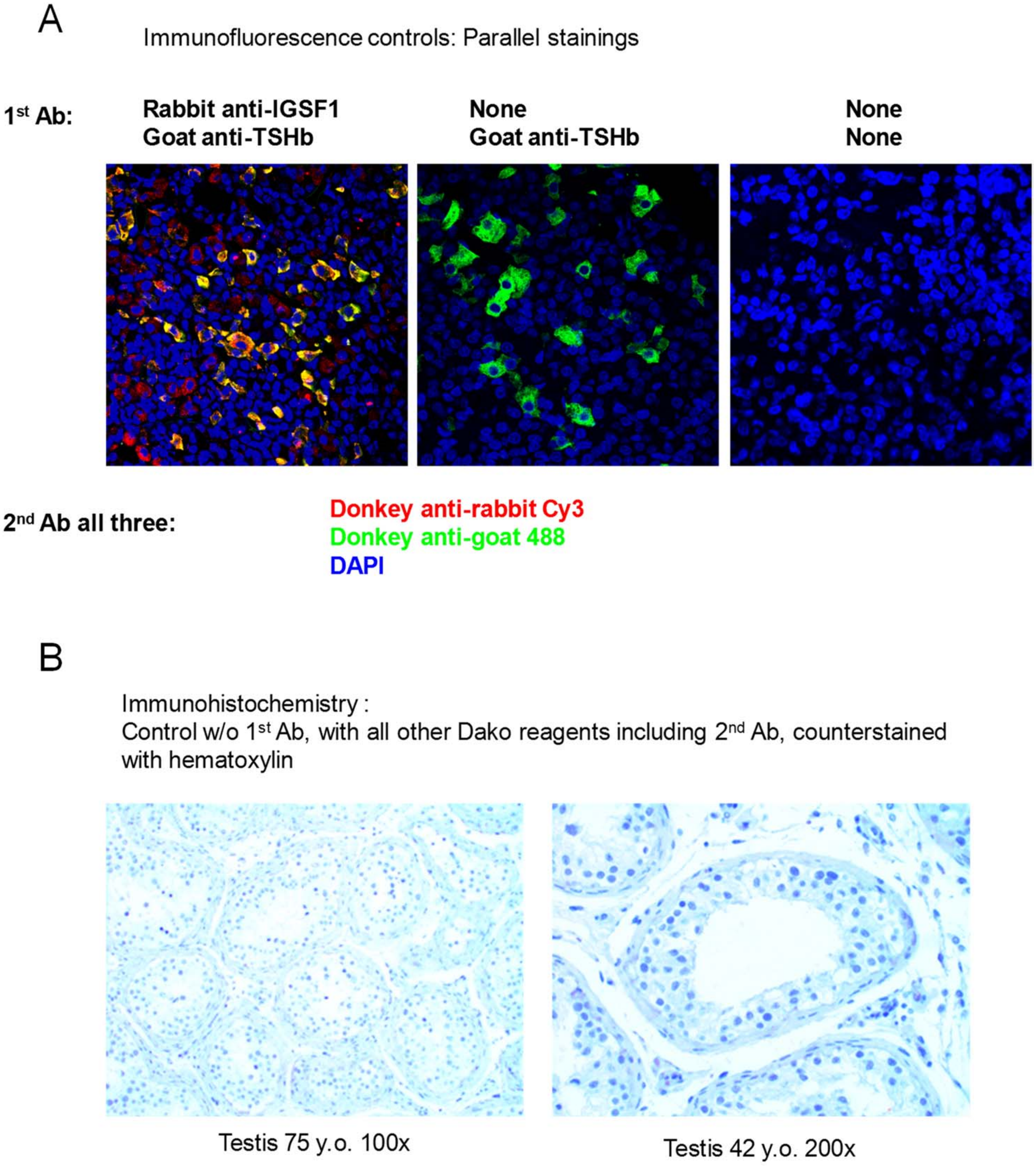

Supplemental Figure 7: Controls of immunostainings

A) Parallel staining of rat pituitary sections for double immunofluorescence with rabbit anti-IGSF1 plus goat-antiTSH (left), omitting the IGSF1 antibody (centre), or with no primary antibody (right). The secondary antibody for all three sections were: donkey anti-rabbit Cy3 (red cells, IGSF1+), donkey anti-goat 488 (green cells, TSH+) together with DAPI.

B) Immunohistochemistry performed in sections of human testes as in Figure 3 but omitting the primary antibody, and also counterstained with hematoxylin.

Hormone Assay	Source	Sensitivity	Range of Detection	Standard calibration
Human TSH	Immulite 2000 analyzer (Siemens Healthcare Diagnostics)	0.001 mIU/L	0.01 -100 mIU/L	NCCLS document EP17
FT4	Vitros ECi immunoanalyzer	0.24 pmol/L	0.88-90 pmol/L	NCCLS document EP17
FT3	Vitros ECi immunoanalyzer	0.18 pmol/L	0.77-35 pmol/L	NCCLS document EP17
Human FSH	Immulite 2000 analyzer (Siemens Healthcare Diagnostics)	0.1 mIU/L	up to 170 mIU/mL	WHO 2nd IRP 78/549
Human LH	Immulite 2000 analyzer (Siemens Healthcare Diagnostics)	0.05 mIU/mL	up to 200 mIU/mL	WHO 1st IRP 68/40 and 2nd IS 80/552
Testosterone	Coat-A-Count radioimmunoassay (Siemens Healthcare Diagnostics)	0.5 nmol/L	0.7 and 55 nmol/L	Δ4-Androsten-17Beta-ol-3-one
Human Inhibin B	OBI-DSL (Beckman Coulter)	7 pg/ml	11-900 pg/ml	Note: 0.5% cross-reaction to Inhibin A
Human AMH	Immunotech SAS	0.02 ng/mL	0.1-22 ng/mL	CLSI EP17-A2

Supplemental Table 1. Analytical limits of detection and dynamic range of the hormone assays

1st Antibody	Source	Origin	Dilution		
			WB	IF	Magnetic immunopurification
IGSF-1	Genetex, GTX112633	R	1:1000	1:200, 4ºC, o.n.	
a-Tubulin	Sigma, T5168	M	1:2500		
aGSU (hCG alpha)	Sunny lab (BM319-MGH2)	M		1:100, 4ºC, o.n.	
GH	NIDDK, AFP222387790	Gp		1:800, R.T., 1h	
GH	NIDDK, AFPC11981A	R			1:25, ice, 30 min.
ACTH	Dako, M3501	M		1:100, 4ºC, o.n.	
PRL	Biodesign, E30610M	M		1:500, 4ºC, o.n.	
Beta-TSH	Santa Cruz Biotech, sc-7813	G		1:50, 4ºC, o.n.	
Beta-TSH	NIDDK, AFP1274789	R			1:12,5, ice, 30 min
Beta-LH	Thermo, Ms-9078P1	M		1:100, 4ºC, o.n.	
Beta-FSH	Zymed, 18-0020	M		1:100, 4ºC, o.n.	
Beta-FSH	NIDDK, AFP7798-1289	R	1:1000		1:12,5, ice, 30 min
Pit-1	BD, 610223	M		1:200, 4ºC, o.n.	
Phospho-SMAD2 Ser465-467	Cell Signaling, 3108	R	1:1000		
SMAD2/3	Santa Curz Biotech, sc-8332	R	1:1000		

2nd Antibody	Company	Origin	Dilution
Anti-rabbit IgG-HRP	*GE Healthcare NA934V*	*D*	*1:5000*
Anti-mouse IgG/HRP	*DAKO, P0260*	*R*	*1:5000*
Anti-mouse Alexa 488<br>Donkey Anti-Mouse IgG (H+L) Alexa Fluor 488	*Molecular Probes, A-21202*	*D*	*1:400*
Anti-rabbit-Fab-Cy3<br>Goat Anti-Rabbit IgG, F(ab')2 Fragment Specific)	*Jackson, 111-166-047*	*G*	*1:600*
Anti-Guinea pig-IgG-Alexa F488<br>Goat Anti-Guinea Pig IgG Alexa Fluor 488	*Molecular Probes, A-11073*	*G*	*1:1500*
Anti-goat-Fab-IgG Cy2<br>Donkey Anti-goat F(ab')2 IgG (H+L) Cy2	*Jackson, 705-226-147*	*D*	*1:600*
Anti-Rabbit IgG MACS MicroBeads	*Miltenyi Biotec, 130-048-602*	*G*	*1:4, ice, 15 min*

Supplemental Table 2. Antibodies and kits used in the experiments and its dilutions. (M=mouse; R=rabbit; G=Goat; Gp=guinea pig; D=Donkey)
